# A Framework of Paracellular Transport via Nanoparticles‐Induced Endothelial Leakiness

**DOI:** 10.1002/advs.202102519

**Published:** 2021-09-08

**Authors:** Myeongsang Lee, Nengyi Ni, Huayuan Tang, Yuhuan Li, Wei Wei, Aleksandr Kakinen, Xulin Wan, Thomas P. Davis, Yang Song, David Tai Leong, Feng Ding, Pu Chun Ke

**Affiliations:** ^1^ Department of Physics and Astronomy Clemson University Clemson SC 29634 USA; ^2^ National University of Singapore Department of Chemical and Biomolecular Engineering 4 Engineering Drive 4 Singapore 117585 Singapore; ^3^ Liver Cancer Institute Zhongshan Hospital Key Laboratory of Carcinogenesis and Cancer Invasion Ministry of Education Fudan University Shanghai 200032 China; ^4^ Drug Delivery Disposition and Dynamics Monash Institute of Pharmaceutical Sciences Monash University 381 Royal Parade Parkville VIC 3052 Australia; ^5^ Key Laboratory of Luminescence Analysis and Molecular Sensing Ministry of Education College of Pharmaceutical Sciences Southwest University 2 Tiansheng Rd, Beibei District Chongqing 400715 China; ^6^ Australian Institute for Bioengineering and Nanotechnology The University of Queensland Brisbane Qld 4072 Australia; ^7^ State Key Laboratory of Environmental Chemistry and Ecotoxicology Research Center for Eco‐Environmental Sciences Chinese Academy of Sciences Beijing 100085 China; ^8^ The GBA National Institute for Nanotechnology Innovation 136 Kaiyuan Avenue Guangzhou 510700 China

**Keywords:** discrete molecular dynamics simulation, endothelial leakiness, signaling pathway, transwell, VE cadherin

## Abstract

Nanomaterial‐induced endothelial leakiness (NanoEL) is an interfacial phenomenon denoting the paracellular transport of nanoparticles that is pertinent to nanotoxicology, nanomedicine and biomedical engineering. While the NanoEL phenomenon is complementary to the enhanced permeability and retention effect in terms of their common applicability to delineating the permeability and behavior of nanoparticles in tumoral environments, these two effects significantly differ in scope, origin, and manifestation. In the current study, the descriptors are fully examined of the NanoEL phenomenon elicited by generic citrate‐coated gold nanoparticles (AuNPs) of changing size and concentration, from microscopic gap formation and actin reorganization down to molecular signaling pathways and nanoscale interactions of AuNPs with VE‐cadherin and its intra/extracellular cofactors. Employing synergistic in silico methodologies, for the first time the molecular and statistical mechanics of cadherin pair disruption, especially in response to AuNPs of the smallest size and highest concentration are revealed. This study marks a major advancement toward establishing a comprehensive NanoEL framework for complementing the understanding of the transcytotic pathway and for guiding the design and application of future nanomedicines harnessing the myriad functions of the mammalian vasculature.

## Graphical abstract

A framework elucidating the paracellular pathway via nanoparticles‐induced endothelial leakiness is presented.

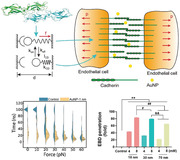

## 1 Introduction

The human vasculature connects the heart with tissues and organs of the body to enable exchange of oxygen and nutrients and elimination of waste via controlled permeability of blood vessels. Recently, it has been found that inorganic nanoparticles can disrupt the vascular endothelial cadherin (VE‐cadherin) junctions of apposing endothelial cells,^[^ ^1^ ^]^ causing actin reorganization and transient gap formation among the impacted cells and tissues, sans cytotoxicity.^[^ ^2^ ^]^ This phenomenon, termed as nanomaterial‐induced endothelial leakiness (NanoEL), is postulated to originate from the physical interactions between exogeneous nanoparticles and the extracellular domains of VE‐cadherin junctions, with allosteric effects exerted on their associated intracellular signaling, regulatory and scaffold machineries such as catenin proteins p120, *β*‐catenin, plakoglobin, and actin.^[^ ^2^, ^3^ ^]^ These biophysical and biochemical characteristics suggest that NanoEL is a statistically quantifiable microscopic phenomenon with a molecular origin, in contrast to the qualitative nature of the enhanced permeability and retention (EPR) effect, a core concept in the field of nanomedicine for describing the adsorption and permeation of nanoparticles in tumor vasculature mediated by non‐specific interactions, local alterations in acidity, as well as blood flow.^[^ ^4^ ^]^

Recent studies have revealed that NanoEL typically occurs on the timescales of sub‐hours to hours upon nanoparticle exposure.^[^ ^2c^ ^]^ Furthermore, it has been identified that the NanoEL‐competent nanoparticles are usually anionic or near neutral in charge and less than 100 nm in size,^[^ ^3^, ^5^ ^]^ which enable them to divert from the transcellular route of endocytosis through charge repulsion and, instead, partition into the paracellular pathway. Toward establishing quantitative descriptors for NanoEL, a concept pivotal to the fields of nanotoxicology and cancer nanomedicine, in the present study we employed synergic in silico, in vitro and ex vivo methodologies to systematically quantify endothelial leakiness associated with citrate‐coated gold nanoparticles (AuNPs) of 18, 30, and 70 nm in size, from the microscopic level down to the nanoscale. AuNPs are a most representative nanomaterial for this study due to their facile synthesis, excellent suspensibility and biocompatibility, and broad biomedical applications,^[^ ^6^ ^]^ Confocal fluorescence microscopy, microtome transmission electron microscopy (m‐TEM), as well as transwell and signaling pathway assays revealed the microscopic features and molecular contributors of leakiness in human microvascular endothelial cell (HMVEC) monolayers exposed to the AuNPs, and further confirmed the co‐existence of endocytosis along the transcellular route. Furthermore, AuNPs translocated across swine vessels, as indicated by a novel ex vivo transwell assay. Using steered discrete molecular dynamics (sDMD) computer simulations, we found that the AuNPs displayed a greater propensity for the extracellular EC1 domain over the EC2 domain of VE‐cadherins. In the presence of an AuNP, the EC1‐EC1 dimer junction yielded to a pulling force within the typical range of 0–30 pN more readily than the control without AuNP binding. Extending the atomistic simulations to coarse‐grained mesoscale simulations of the cell–cell junction consisting of two membranes stabilized by an array of cadherin dimers, NanoEL was found to nucleate by the dissociations of cadherin dimers bound with AuNPs under a tensile force, which subsequently propagated to adjacent cadherin dimers through breaking the dissociation‐rebinding balance. Localized AuNPs in the narrow paracellular route could further enhance endothelial leakiness.

Together, this comprehensive study offered a new framework for quantifying NanoEL in silico, in vitro and ex vivo, representing a crucial advancement toward deciphering and harnessing the paracellular pathway of the vasculature, which has been largely overlooked so far in favor of the transcytotic pathway in nanomedicine and nanotoxicology.

## 2 Results and Discussions

### 2.1 Characterizations of AuNPs

The primary sizes and *ζ*‐potential values of AuNPs of 18, 30, and 70 nm (in hydrodynamic diameter) are presented in Figure S1A and Table S1 (Supporting Information), based on consistent transmission electron microscopy and dynamic light scattering measurements in ultrapure water and complete cell medium. The hydrodynamic diameter of the three sizes of AuNPs in complete cell medium increased due to the formation of protein coronae on the surface of the AuNPs. The *ζ*‐potential values of all AuNPs samples were negative, within the range of −26.7 to −41.7 mV in ultrapure water and −12.5 to −15.4 mV in complete cell medium, further indicating good suspensibility of the AuNPs. The peak absorbance of the AuNPs showed a redshift with the increasing size due to dampened surface plasmon resonance (Figure S1B, Supporting Information). There was no generation of reactive oxygen species (ROS) over a time course of 120 min for HMVECs exposed to the AuNPs of three sizes and two concentrations (25, 100 × 10^−6^ m) (Figure S1C, Supporting Information).

### 2.2 NanoEL Characterized In Vitro with Confocal Fluorescence Microscopy

**Figure 1:**
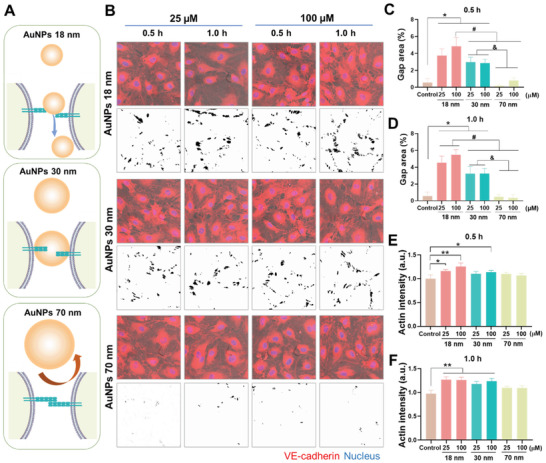
AuNPs‐induced endothelial leakiness and actin reorganization in HMVECs. A) Illustrated interactions between adherens junction and AuNPs of different sizes. VE–cadherin homophilic interactions among endothelial cells assist the connection and stability of the intact monolayer. The introduction of the different sized AuNPs affected the integrity of the adherens junction to different extents. The 18 and 30 nm AuNPs were small enough to migrate into the adherens junction and disrupt VE–cadherin homophilic interaction, while the 70 nm AuNPs were not able to cause significant disruption. B) Confocal fluorescence microscopy observed the occurrence of endothelial leakiness in the presence of different sizes (18, 30, and 70 nm) and concentrations (25 and 100 × 10^−6^ m) of AuNPs upon 0.5 and 1 h treatments. The images with black dots on a white background revealed the gaps’ distributions derived from the trainable Weka segmentation plugin in ImageJ software. Scale bar: 20 µm. C,D) Percentages of gaps area analyzed by ImageJ according to the gaps’ distribution images from panel B. E,F) Actin intensity analysis was performed by ImageJ software for the images in Figure S5 (Supporting Information). Data are shown as mean ± SD (*n* = 3), analyzed via two‐way ANOVA using GraphPad Prism 8, *^, #,^ *^&^* represent *P* < 0.01 and ** represents *P* < 0.001 between the compared groups.

Confocal fluorescence imaging revealed the occurrence of endothelial leakiness in HMVECs incubated with the AuNPs of three sizes (**Figure** **1**). The extent of NanoEL was determined by the gap area and distribution analyses (Figure 1B–D and Figure S2: Supporting Information). The 18 nm AuNPs induced the maximum extent of NanoEL followed by the 30 nm AuNPs and then the 70 nm AuNPs. Specifically, with the concentration of the 18 nm AuNPs increased from 25 to 100 × 10^−6^ m, the percentage of gap area was elevated from 3.75 ± 0.80% to 4.85 ± 1.03% at 0.5 h and from 4.55 ± 0.77% to 5.48 ± 0.60% at 1 h (Figure 1C,D and Table S2: Supporting Information). In addition, the number of gaps induced by the 18 nm AuNPs rose from 27.0 ± 6.2 × 10^2^ to 49.6 ± 6.2 × 10^2^ gaps mm^−2^ at 1 h of exposure (Figure S3, Supporting Information). The extent of endothelial leakiness induced by the 30 nm AuNPs was notably less than that for the 18 nm AuNPs, while the 70 nm AuNPs exerted little effect on the integrity of the endothelial monolayer. The extracellular (EC) domains of the VE‐cadherin dimer (Figure S4, Supporting Information) stabilizing the adherens junctions had the end‐to‐end distance of ≈36 nm. Accordingly, NPs larger than 36 nm encountered a greater difficulty to enter and be aligned to disrupt the homophilic interactions of VE‐cadherins. In addition, concentration (25 and 100 × 10^−6^ m) and processing time (0.5 and 1 h) appeared to have a smaller effect on the extent of NanoEL in the group of 30 nm AuNPs, different from the AuNPs of 18 and 70 nm. Concomitantly, the cytoskeletal actin network appeared reorganized in conjunction with the NanoEL phenomenon, based on the altered fluorescence intensity of phalloidin‐iFluor 488 and the spatial re‐arrangement of actin filaments, where most significant actin reorganization was observed for AuNPs of 18 nm and 100 × 10^−6^ m at 0.5 h (Figure 1E and Figure S5: Supporting Information). In comparison, the actin intensity was significantly greater induced by AuNPs of 18 and 30 nm at both 25 and 100 × 10^−6^ m after 1 h of treatment (Figure 1F and Figure S5: Supporting Information).

### 2.3 NanoEL Characterized In Vitro with Microtome Electron Microscopy

**Figure 2:**
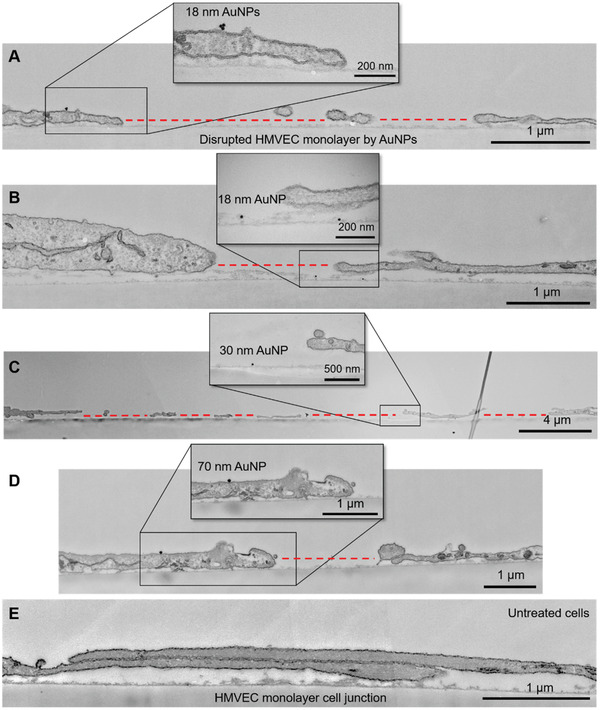
Microtome transmission electron microscopy imaging of endothelial leakiness in HMVEC monolayers induced by AuNPs. A,B) Disruption of HMVEC monolayers by AuNPs of 18 nm in size after 30 min of exposure. Panel A shows the presence of an AuNP on the edge of cell membranes adjacent to the EL site. Panel B shows the presence of an AuNP inside the EL gap between two cells. C) EL between HMVECs induced by AuNPs of 30 nm in size. D) EL induced by AuNPs of 70 nm in size. E) Cell junctions between two HMVECs (control). The insets of panels A–D are zoomed in sections showing the presence of AuNPs. The EL sites are indicated by red dotted lines.

To reveal the cellular state upon the occurrence of NanoEL at higher resolution than confocal fluorescence microscopy, we performed microtome transmission electron microscopy (mTEM) of HMVECs exposed to the AuNPs (**Figure** **2**). Gaps of 3.1 ± 1.5 µm in size were found for HMVECs incubated with the three sized AuNPs for 30 min. While the appearance of AuNPs was scarce due to repeated washing procedures with mTEM, we still spotted AuNPs (Figure 2A–D) and occasionally noticed small traces of 18 nm AuNPs endocytosed within the intracellular space in endosomes/lysosomes (Figure S6, Supporting Information). Notably, no endocytosis was observed for AuNPs of 30 and 70 nm within 30 min of exposure.

### 2.4 NanoEL In Vitro Independent of Nanotoxicity Descriptors

**Figure 3:**
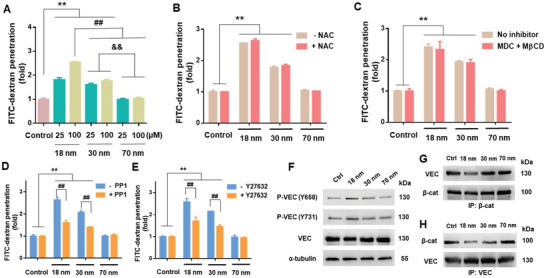
Endothelial leakiness induced by AuNPs was dose‐dependent but endocytosis‐independent, and required activation of VE‐cadherin signaling. A) Transwell assay revealed dependence of NanoEL on the size and concentration of the AuNPs (incubation: 1 h). B) No significant differences occurred in FITC‐dextran penetration across endothelial barriers exposed to AuNPs (100 × 10^−6^ m, 1 h) with or without prior antioxidant NAC (10 × 10^−3^ m) treatment (1 h). C) Endocytosis inhibitors (5 × 10^−3^ m M*β*CD and 10 × 10^−6^ m MDC) supplied 1 h prior to AuNP treatment (100 × 10^−6^ m) did not result in a significant reduction in FITC‐dextran penetration. D) Degree of NanoEL induced by AuNPs (100 × 10^−6^ m, 1 h) was significantly reduced through pre‐treatment with Src kinase inhibitor, PP1 (10 × 10^−6^ m, 1 h). E) Pre‐treatment with RhoA kinase inhibitor Y27632 (10 × 10^−6^ m, 1 h) led to a significant reduction in NanoEL induced by AuNPs (100 × 10^−6^ m, 1 h). F) Immunoblotting revealed activation of VE‐cadherin (VEC) signaling when exposed to the AuNPs (100 × 10^−6^ m, 1 h). Phosphorylation of VEC at residues 658 (P‐VEC(Y658)) and 731 (P‐VEC(Y731)) increased with decreasing size of AuNPs. G,H) Immunoprecipitation of *β*‐catenin (*β*‐cat) revealed decreased interactions between VEC and *β*‐cat following cell exposure to the AuNPs (100 × 10^−6^ m, 1 h). The reverse immunoprecipitation of VEC similarly revealed a decreased association between VEC and *β*‐cat. Data are presented as mean ± SD, where *n* = 3, analyzed via two‐way ANOVA with Tukey's multiple comparison tests, and **^, ##, &^ *^&^* all denote *P* < 0.001 between the various compared groups.

To reveal the cellular mechanisms pertinent to the NanoEL phenomenon, we examined common contributors to intercellular gap formation. Shrinkage of cells due to apoptosis,^[^ ^7^ ^]^ such as via the generation of ROS,^[^ ^8^ ^]^ has been established in nanotoxicology.^[^ ^9^ ^]^ We proceeded with cell viability, membrane damage and ROS production detection of endothelial cells subjected to AuNPs of 18, 30, and 70 nm, for concentrations of 25 and 100 × 10^−6^ m and at exposures of 1 and 6 h. The profiles did not reveal significant changes in cell viability, membrane damage or increase in ROS production up to 6 h (Figure S7, Supporting Information). We then employed transwell assays to quantify the AuNPs‐induced NanoEL phenomenon with a fluorescent probe, fluorescein isothiocyanate conjugated dextran (FITC–dextran), to gauge the degree of leakiness in an endothelial barrier. We observed an increased penetration of FITC‐dextran across the endothelial barrier with decreasing size of the AuNPs (**Figure** **3**A), where the largest 70 nm AuNPs showed a negligible FITC‐dextran transport. HMVECs pretreated with antioxidant N‐acetyl cysteine (NAC) 1 h prior to AuNPs exposure could not alleviate FITC‐dextran transport either, revealing a minimal role of oxidative stress in NanoEL (Figure 3B). Together, these observations corroborated our earlier findings with TiO_2_ nanoparticles, where NanoEL did not result from a decline in cell health or ROS generation.^[^ ^2a^ ^]^

### 2.5 NanoEL Characterized In Vitro by Molecular Signaling Pathways

With the elimination of key intracellular potential contributors, we further examined the potential role of NanoEL occurring due to the interactions of AuNPs with cell membranes. In Figure 2 and Figure S6 (Supporting Information), captured mTEM images revealed the presence of AuNPs at the endothelial cell junctions, as well as small traces of 18 nm AuNPs which were endocytosed. To confirm that the association of AuNPs with cell junctions played a key role in NanoEL, but not the internalization of the AuNPs, we employed a cocktail of endocytosis inhibitors (5 × 10^−3^ m methyl‐*β*‐cyclodextrin (M*β*CD) and 10 × 10^−6^ m monodansycadaverine (MDC), which ascertained that blocking endocytosis could not alleviate the NanoEL effect associated with the AuNPs (Figure 3C). These results were consistent with our early observations, validating the onset of NanoEL was a result of extracellular triggering.^[^ ^3^ ^]^

Our previous work revealed that NanoEL occurred due to NPs disrupting VE‐cadherin homophilic interactions at the adherens junction, with activation of VE‐cadherin signaling.^[^ ^2a^ ^]^ Toward a molecular understanding and quantification of the NanoEL phenomenon, we therefore further investigated the activation of VE‐cadherin signaling through phosphorylation of its two pivotal residues, tyrosine 658 (Y658) and tyrosine 731 (Y731) in HMVECs. Phosphorylation at Y658 is further known to lead to the internalization of VE‐cadherin and its subsequent proteolytic degradation.^[^ ^10^ ^]^ In agreement with our earlier work with TiO_2_ nanoparticles,^[^ ^2a^ ^]^ AuNPs also led to significant phosphorylation at Y658 and Y731 (Figure 3F). The degree of phosphorylation correlated inversely with the size of the AuNPs (Figure S8A,B: Supporting Information). Overall VE‐cadherin in HMVECs was also most significantly reduced for the 18 nm AuNPs group, compared with control (Figure S8C, Supporting Information). To affirm the involvement of the two pivotal residues in AuNPs‐induced endothelial leakiness, PP1, an inhibitor that blocks the Src kinase involved in their phosphorylation, was utilized. In our transwell assay, PP1 treatment revealed a significant alleviation but not complete inhibition of the induced leakiness in endothelial barriers (Figure 3D). This further supported the involvement of VE‐cadherin activation in the endothelial leakiness induced by AuNPs.

In addition, phosphorylation at Y731 residue has been known to lead to the loss of endothelial barrier function through disrupting interactions between *β*‐catenin and VE‐cadherin proteins, which further lead to the loss of interactions with and subsequent remodeling of the actin cytoskeleton.^[^ ^11^ ^]^ In our immunoprecipitation assays, employing *β*‐catenin as a precipitant revealed a reduced association with VE‐cadherin for treatment with AuNPs of decreasing sizes (Figure 3G and Figure S8D: Supporting Information). A complementary assay, with VE‐cadherin as the precipitant, revealed a similar trend (Figure 3H and Figure S8E: Supporting Information). To demonstrate the importance of actin remodeling in NanoEL with AuNPs, we utilized a RhoA kinase (ROCK) inhibitor, Y27632, which is known to reduce the formation of stress fibers and destabilize focal adhesions, thus disrupting the normal function of actin remodeling.^[^ ^12^ ^]^ Transwell assays indicated that such disruption led to a suppression of NanoEL, thus implicating a role of the cytoskeletal network in the molecular mechanism of NanoEL (Figure 3E). Collectively, Figure 3 validated that intracellular signaling in NanoEL lied in the interactions between AuNPs and VE‐cadherins, with the smallest AuNPs giving rise to the greatest activation of adherens junction.

### 2.6 NanoEL Characterized Ex Vivo with Swine Vessels

**Figure 4:**
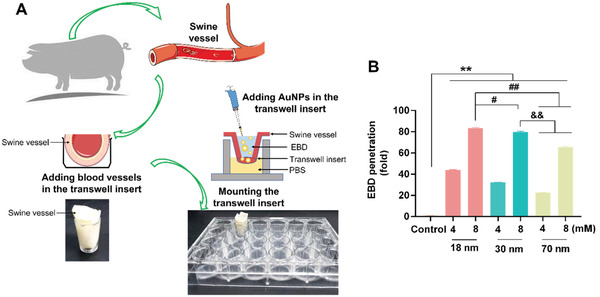
AuNPs‐induced endothelial leakiness in swine vessels. A) Scheme of the *ex vivo* construct. B) Size‐dependent decrease in the leakiness of swine vessels, consistent with the in vitro experiments. The degree of Evans blue dye (EBD) penetration in swine vessels was inversely proportional to the size of the AuNPs, for both concentrations of 4 and 8 × 10^−3^ m, showing significant differences compared with the untreated control. EBD penetration was more pronounced in the 18 nm AuNPs group (8 × 10^−3^ m). Quantification of EBD showed more leakiness in the 18 nm AuNPs group compared with the 30 and 70 nm AuNPs groups. Results represent for means ± SD (*n* = 3), via one‐way ANOVA analyzed for 18, 30, and 70 nm AuNPs at 4 and 8 × 10^−3^ m treatments, ^#^ *P* < 0.01, **^, ##,^ *^&&^P* < 0.001.

The in vitro observations above were corroborated with an ex vivo assay to characterize the leakiness of swine vessels. We noted that the highest degree of swine vessels leakiness occurred with AuNPs of 18 nm in size and 8 × 10^−3^ m in concentration (**Figure** **4**). The extents of endothelial leakiness induced by the 30 nm AuNPs and 70 nm AuNPs were notably less than that for the 18 nm AuNPs, while the 70 nm AuNPs elicited the lowest leakage. In addition, concentration appeared to be a major factor in the extent of NanoEL, where the degree of vessel leakiness was significantly greater induced by AuNPs of all three sizes at 8 × 10^−3^ m than at 4 × 10^−3^ m. These results are in good agreement with the in vitro data in Figures 1 and 3.

### 2.7 Molecular VE‐Cadherin Dimer Dissociation Characterized with In Silico Force Microscopy

**Figure 5:**
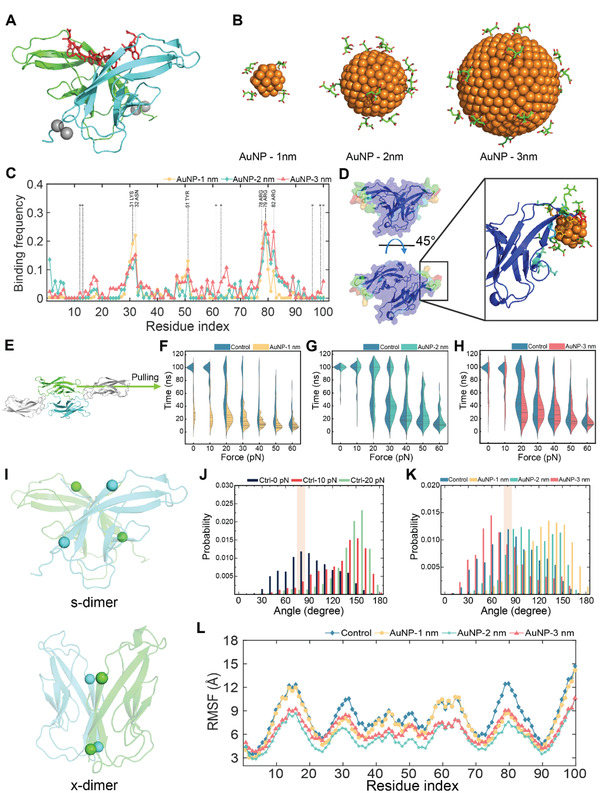
Schematic of the EC1 cadherin dimer and AuNPs considered for this study, their corresponding binding frequency, as well as effects of the AuNPs on the EC1 cadherin dimer stability. A) Structure of the EC1 cadherin dimer. The domain‐swapped region and calcium ions are represented as red sticks and silver spheres, respectively. B) Different sized AuNPs coated with citric acids. C) Binding frequency of the AuNPs with the EC1 cadherin dimer. The residues coordinating the calcium ions in the EC1 dimer were highlighted by star (*). D) Binding frequency with the 1 nm AuNP colored on the surface of the EC1 cadherin dimer. Blue and red represent low to high binding frequencies, respectively. Enlarged panel illustrates details of AuNP binding with the EC1 dimer. E) Schematic of the sDMD simulation. The flexible domain and immoblilized domain from EC1 were colored in green and cyan, respectively. EC2 domains were colored in gray. F–H) Force dynamics for the EC1 dimer with the 1, 2, and 3 nm AuNP, respectively. I) Two different states of the EC1 cadherin dimer as the domain swapped structure known as the s‐dimer (top) and the intermediate state as the x‐dimer (bottom). Four C*α* residues represented as spheres were overlaid to represent the two vectors of the G‐strands of the immunoglobulin folds in the EC1 dimer for calculating the EC1 dimer angle. J) EC1 dimer angle distribution without an AuNP under applied forces of 0, 10, and 20 pN. K) EC1 dimer angle distribution under 0 pN with and without an AuNP. The dimer angles of the s‐dimer in J&K were shaded as rectangular boxes. L) Root mean square fluctuation (RMSF) of the flexible domains of the EC1 cadherin dimer.

To understand the destabilization of VE‐cadherin dimer by AuNPs, sDMD simulations using atomistic models with an implicit solvent were applied first to characterize the force‐induced dissociation of a VE‐cadherin dimer in the presence of AuNPs. Before exerting pulling forces to the VE‐cadherin dimer in silico, the initial binding of AuNPs with the dimer was determined using equilibrium DMD simulations. In the full‐length of VE‐cadherin dimer, the EC1 domains formed a domain‐swapped dimer that are important for the trans cell‐to‐cell adhesion (**Figure** **5**A). Therefore, we considered only the EC1 dimer in the following simulations. Due to the high computational cost associated with large nanoparticles, we considered citric acid‐coated AuNPs of 1, 2, and 3 nm in diameter (Figure 5B) in our atomistic simulations to determine the location and mode of AuNPs binding with the EC1 cadherin dimer and to demonstrate both the quantitative and qualitative nature of the NanoEL phenomenon in silico.

Starting with the AuNPs randomly positioned away from the dimer, multiple independent simulations at 300 K were performed to examine the binding of the AuNPs with the EC1 dimer. After reaching equilibrium, we calculated the binding frequencies of the AuNPs with residues in the EC1 dimer (Figure 5C). AuNPs preferred to bind residues 29–32 and 78–82 in the EC1 cadherin dimer, corresponded to the loop regions away from the dimer interface as demonstrated by the surface representation of the dimer with each residue colored according to their AuNP‐binding frequency (Figure 5D). The high binding residues in the loops were mostly positively charged (Figure 5C), forming favorable electrostatic interactions with the citrate‐coated AuNPs. The EC1 structure adopted the immunoglobulin (Ig) fold, featuring two loop regions on the opposite sides of the *β*‐sandwich. The AuNP‐binding loops were away from the calcium‐binding loops (Figure 5C), due to the negatively charged residues that formed coordination bonds with the ions.^[^ ^1a^ ^]^ It is interesting to note that calcium binding is important for stabilizing the cadherin dimer and thus the endothelial cell–cell junction.^[^ ^1^, ^13^ ^]^ We additionally computed the binding frequency of the AuNPs with the EC12 cadherin dimer to identify whether the AuNPs had any binding preference to the EC1 and EC2 domains (Figure S9, Supporting Information). All AuNPs were found to preferentially bind to the EC1 domain in the EC12 cadherin dimer, likely because the equivalent loops for AuNP‐binding in the EC2 domain were in the proximity of the calcium‐binding loops in EC1. Hence, AuNPs dominantly bound to the EC1 domain of VE‐cadherin and the binding to VE‐cadherin was primarily driven by electrostatic interactions.

Next, we performed sDMD simulations to study the effects of AuNP‐binding on the EC1 dimer stability. Constant forces in a range of 0–60 pN, experimentally shown to dissociate or disrupt the various species of cadherin dimers,^[^ ^1^, ^14^ ^]^ were applied to the center of one of the EC1 domains and along the direction from EC1 to EC2, while the other EC1 domain was kept immobile (Figure 5E and Experimental Section). For each constant pulling force, 30 independent simulations were performed with randomized initial velocities assigned according to Maxwell‐Boltzmann distribution. sDMD simulations in the absence of NPs were also performed for comparison. For each pulling simulation, we computed the dissociation time of EC1 cadherin dimer, defined as the time when the number of inter‐chain contacts of the EC1 cadherin domains was reduced to zero, and represented them as a function of applied forces in the violin plots (Figure 5F–H). In cases where the dimer did not dissociate during the entire course of the simulations, the dissociation time was assigned as the maximum simulation time of 100 ns. With increasing forces, the dimer was dissociating at short times with high probabilities. In the presence of AuNPs of all three sizes, shorter dimer dissociation times were generally observed with higher probabilities compared with the controls under the same pulling forces. In particular, the 1 nm AuNP showed the highest cadherin dimer dissociation under the low force range (0–30 pN) compared to the 2 and 3 nm AuNPs. Although the AuNPs used in the sDMD simulations were not of the same sizes as in the experiments, the observed stronger cadherin dissociation by AuNPs of smaller sizes was consistent with experiments (Figures 1B,C and 4). We noted that the dimer dissociation was a highly stochastic process and an accurate estimation of the mean dissociation time or the corresponding off‐rate governed by the dissociation free‐energy barrier might require significantly more independent sDMD simulations than 30, which were limited by available computational resources. Nevertheless, our sDMD simulations confirmed that AuNPs destabilized the VE‐cadherin dimer by binding near the ion‐coordinating loops away from the dimer interface.

It has been suggested that the domain‐swapped cadherin dimer (i.e., the s‐dimer) is formed via an intermediate state without domain‐swapping, namely the x‐dimer.^[^ ^1^, ^13^ ^]^ The x‐dimer intermediate has been observed in X‐ray crystallography, which features a different inter‐domain orientation from the s‐dimer (Figure 5I). To understand AuNP binding‐induced destabilization of the VE‐cadherin dimer, we measured an angle between the two vectors in each EC1 domain from residues 88 to 95 along a G‐strand near the dimer interface (Figure 5J,K). The x‐dimer featured an approximately parallel alignment of the two strands with an inter‐domain angle of ≈0.5° compared to ≈85° for the s‐dimer with two strands in perpendicular.^[^ ^1^, ^15^ ^]^ In the absence of AuNPs, we computed the distribution of inter‐domain angles of the dimer before dissociation under 0, 10, and 20 pN external pulling forces (Figure 5J). With increasing forces, we did observe the equilibrium shifted away from the s‐dimer like structures toward anti‐parallel alignments, resulting in shorter dissociation times (Figure 5F). Hence, our results suggested that structures with the parallel or anti‐parallel alignments were less stable compared to the s‐dimer due to induced strains in the dimeric state. Next, we compared the distribution of inter‐domain angles in the presence of the AuNPs under 0 pN force (Figure 5K). Compared to the control simulations without AuNPs at 0 pN, the 1 and 2 nm AuNPs shifted the angle distribution to the large values of ≈160–180°, while the 3 nm AuNP altered the distribution to a lower value of ≈50–60° (typical snapshots shown in Figure S10: Supporting Information). Hence, the AuNP‐binding shifted the equilibrium of the EC1 cadherin dimer from the stable s‐dimer to the less stable states with altered alignments between the two domains, thereby promoting dissociation. We also calculated the root mean square fluctuation (RMSF) of the EC1 cadherin dimer with and without the AuNPs (Figure 5L). RMSF was measured for the first 30 ns by sDMD simulation before the EC1 cadherin dimer underwent dissociation. All AuNPs significantly reduced the conformational flexibility and hence the entropy of the EC1 dimer, including regions with weak or no AuNP‐binding. Together, our results revealed that AuNP‐binding destabilized the dimer, by not only disrupting the native interaction at the cadherin dimer interface but also reducing the conformational entropy of the bound state. Our sDMD simulation results were qualitatively consistent with the in vitro and ex vivo data (Figures 1, 2, 3, 4) and offered a molecular insight into the enhanced VE‐cadherin dimer dissociation by AuNPs.

### 2.8 Mesoscopic VE‐Cadherin Dimer Dissociation Characterized In Silico

**Figure 6:**
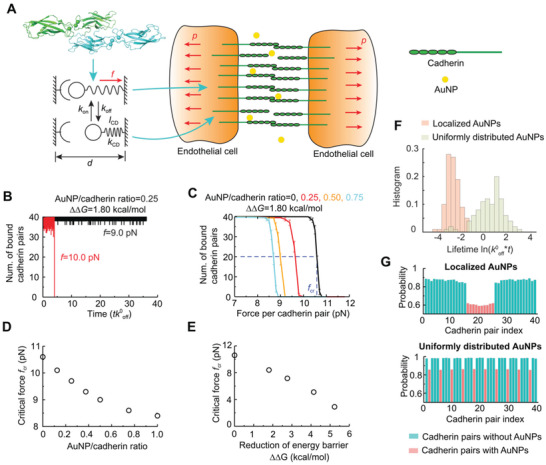
Mesoscale modelling of cadherin pair dissociation in the presence of AuNPs. A) Schematic illustration of the mesoscale model. Left top panel: atomic structure of a cadherin dimer (PDB code: 3PPE); Left bottom panel: a single cadherin pair in bound and unbound states. Right panel: a mesoscale model of endothelial cells connected by arrays of cadherin pairs was subjected to uniform tensile stress *p* in the presence of AuNPs. B) Number of bound cadherin pairs as a function of the rescaled simulation time when AuNP/cadherin ratio was 0.25 and reduction of free energy barrier ΔΔ*G =* 1.80 kcal mol^−1^. C) Number of bound cadherin pairs as a function of tensile force with various AuNP/cadherin ratios. D) Critical rupture force as a function of AuNP/cadherin ratio with ΔΔ*G =* 1.80 kcal mol^−1^. E) Critical rupture force as a function of ΔΔ*G*. The AuNP/cadherin ratio was fixed as 1.0. F) Histogram of cadherin pair cluster lifetime when AuNPs were localized, or uniformly distributed among cadherin pairs. The tensile force was *f* = 10 pN. G) Time‐averaged probability of each cadherin pair in bound state when AuNPs were localized (upper panel) or uniformly distributed among cadherin pairs (bottom panel).

Next, we devised a coarse‐grained model with numerical simulations to offer a mesoscale statistical understanding of the NanoEL phenomenon for the first time (**Figure** **6**A). The details of the mesoscale model can be found in the Experimental Section. Briefly, the cell–cell junction was modelled by two neighboring membranes stabilized by an array of cadherin dimer pairs and the cooperative effect of multiple cadherin pairs was included by considering the dissociation and rebinding of a cadherin cluster under a tensile force (Figure 6A).^[^ ^16^ ^]^ AuNP‐binding destabilized the dimer by increasing the dissociation rate. Evolution of cadherin pair states (e.g., bound or unbound) was simulated by Monte Carlo‐based first reaction method.

We began with the dynamic evolution of the cadherin pair states. The number of bound cadherin pairs as a function of the simulation time with a reduction of free energy barrier induced by AuNPs ΔΔG = 1.80 kcal mol^−1^ is shown in Figure 6B. The AuNPs were randomly distributed with the AuNP/cadherin ratio fixed at 0.25. Under a tensile force *f* = 9 pN per cadherin pair, the cadherin pairs dissociated and rebound frequently with the total number of bound pairs fluctuating around 38, and thus, the junction stayed intact. Under a slightly larger tensile force *f* = 10 pN, however, the cadherin pairs system became unstable and all pairs dissociated in an all‐or‐none manner, resulting in the loss of the junction. The all‐or‐none failure mode of the cadherin cluster agreed well with prior results acquired by atomic force microscopy.^[^ ^17^ ^]^ The evolution of the cadherin pair states showed that rupture of the entire cadherin cluster was initiated by the irreversible dissociation of adjacent cadherin pairs under a large tensile force (Figure S11, Supporting Information). Combining the simulation results with various tensile forces, the average number of bound cadherin pairs as a function of applied force was obtained (Figure 6C), which exhibited a typical sigmoidal characteristic. The critical force to dissociate the junction f_cr_ was defined as the force with half of the cadherin pairs remaining bound in the simulations. In the absence of AuNPs, the critical force was about 10.6 pN, which was on the same order as with the sDMD simulations (Figure 5). The critical force decreased with the increasing AuNP/cadherin ratio (Figure 6D), consistent with the experimental results that a greater extent of endothelial leakiness occurred for AuNPs of higher concentrations (Figures 1B and 4). When all the cadherin pairs were bound with AuNPs which lowered the free‐energy barrier by ΔΔG  = 1.80 kcal mol^−1^, the critical force reduced to 8.4 pN. In addition, to model the size‐dependent effect of AuNPs in facilitating the dissociation of cadherin pairs, the reduction of the free‐energy barrier ΔΔG was varied with the AuNP/cadherin ratio fixed at 1.0, and the results are shown in Figure 6E. The critical force dropped significantly with an increased reduction of the free‐energy barrier induced by AuNPs. When the reduction of the free‐energy barrier induced by AuNPs was 5.23 kcal mol^−1^, the critical force decreased to about 2.9 pN, indicating the dissociation of the cadherin pair cluster and subsequent disruption of the cell–cell junction were effectively facilitated.

The paracellular route where AuNPs transport across the endothelial cell barrier through the intercellular space is narrow and obstructed by various junctional proteins, which could lead to non‐uniform distribution of AuNPs. To investigate the effect of AuNP distribution on the dissociation of the cadherin pair cluster, we considered two specific cases where AuNPs were localized near the center or uniformly distributed among the cadherin pair cluster. Under the force of 10 pN per cadherin, the lifetime of cadherin pairs with uniformly distributed AuNPs was significantly longer than these with localized AuNPs (Figure 6F). The time‐averaged probability of each cadherin pair in the bound state showed that cadherin pairs with localized AuNPs were more likely to dissociate than these with uniformly distributed AuNPs (Figure 6G). The evolution of cadherin pair states and deformation models with concentrated AuNPs (Figure S12, Supporting Information) indicated that localized dissociation of cadherin pairs induced large separations in the vicinity, which subsequently enlarged the tensile force and lowered the rebinding rate between nearby pairs. Thus, the crack propagated rapidly through the entire domain. In comparison, the dissociations of cadherin pairs were dispersed in the cluster domain with uniformly distributed AuNPs (Figure S13, Supporting Information). As a result, it took a longer time to dissociate adjacent cadherin pairs to nucleate defects large enough for further propagation. In summary, our simulations showed that localized AuNPs were more effective in dissociating the cadherin pair cluster. Such knowledge is a first in the field of NanoEL.

## 3 Conclusion

The NanoEL concept is a cornerstone for describing the transport and fate of nanoparticles in endothelial and/or tumoral environments. In comparison with the well‐known EPR effect, NanoEL is a nascent field and much of its framework remains to be established. In the present study, we performed a first of its kind, systematic, top‐down examination to reveal and quantify the intracellular‐extracellular molecular contributors, the biochemical‐biophysical mechanisms, as well as the microscopic‐nanoscale manifestations of the NanoEL phenomenon associated with AuNPs of changing size and concentration. We found that the smallest AuNPs at the highest concentration elicited the strongest response and leakiness in HMVECs (in vitro; Figures 1 and 3 and Figure S8: Supporting Information) and in swine vessels (ex vivo; Figure 4), resulting from the most significant disruptions to the extracellular VE cadherin junction (in silico; Figures 5 and 6) and their intracellular cofactors and molecular assemblies (in vitro; Figure 3). While the EC1 cadherin dimer could disengage more readily in the presence of AuNPs (Figures 3, 4, 5, 6), as an activation‐associated (with energy barriers) phenomenon (Figures 3 and 6) the cadherin junctions could reanneal upon the removal of external mechanical stimuli (Figure 5F–H). This indicated the reversible and kinetic nature of NanoEL that is unknown for the EPR effect, with the latter being a paradigm derived primarily for tumor environments.^[^ ^18^ ^]^ Within this study, for example, there was no co‐culture of cancer cells making NanoEL a nano‐centric paracellular phenomenon distinct from the EPR effect that has been extensively studied and summarized in the literature.^[^ ^2^, ^4^, ^19^ ^]^ In light of the foundational significance of the NanoEL concept for elucidating the nano‐bio interface that is ubiquitous across the fields of nanotoxicology, cancer nanomedicine, and bioengineering, we have come to postulate that the greater and often more desirable biological‐barrier translocation and biodistribution of smaller nanoparticles may originate from their more robust endothelial permeability, in addition to the other factors known as nanoparticle physicochemical properties and self‐agglomeration as well as opsonization of the biological host. To that end, this current study may prove essential for establishing a NanoEL framework for complementing our knowledge on the EPR effect and the transcytotic pathway, thereby facilitating the design of better nanomedicines.

## 4 Experimental Section

### Synthesis of AuNPs

Citrate‐coated AuNP seeds were synthesized by step‐wise seeded growth Turkevich‐Frens protocol^[^ ^20^ ^]^ with slight modifications. Aqueous chloroauric acid (HAuCl_4_, 150 mL, 0.25 × 10^−3^ m, Sigma Aldrich) was brought to boil at 100°C for 15 min under continuous stirring, then sodium citrate solution (Na_3_C_6_H_5_O_7_, 38.75 × 10^−3^ m, Sigma Aldrich) was added to initiate the reduction reaction. Approximately 2.9 and 4.8 mL of Na_3_C_6_H_5_O_7_ solutions were added to render AuNP seeds of 13 and 18 nm, respectively. The solution was kept in boiling condition with vigorous stirring until a wine‐red colored solution appeared. The resultant Au seed solution was kept at room temperature and used for AuNP formation. The AuNPs of 13 nm were used as seeds to produce AuNPs of 30 nm, and the 18 nm AuNPs were used to prepare AuNPs of 70 nm. Thereafter, the as‐synthesized 13 nm AuNPs seeds of 1.6 mL was added into 23.1 mL MilliQ water. Freshly prepared hydroxylammonium chloride (NH_2_OH · HCl, 40 × 10^−3^ m, 0.3 mL) was added to the mixture and then 5 mL of HAuCl_4_ (1 × 10^−3^ m) was added in a dropwise manner. The reaction was maintained in stirring conditions for another 2 h at room temperature to render the 30 nm AuNPs. The 18 nm AuNPs of 0.4 mL were employed to produce the AuNPs of 70 nm according to the same process. The AuNPs were dialyzed against MilliQ water and then kept at 4 °C.

### Characterization of AuNPs

For TEM imaging of the AuNPs, 5 µL of the samples were pipetted on carbon‐coated copper grids (400 mesh, formvar film, ProSciTech). After 60 s of absorption, an excess sample was drawn off using filter paper and grids were washed once with 10 µL of Milli‐Q water. The samples were imaged on a HITACHI HT7700 transmission electron microscopy operated at 80 kV. ImageJ (FIJI) software was used to analyze the images. The absorbance of the AuNPs was obtained by spectra scanning from wavelength of 400 to 800 nm with a UV‐VIS spectrophotometer (UV‐3600, Shimadzu). The hydrodynamic diameter and ***ζ*‐**potential of the AuNPs were determined by dynamic light scattering (DLS) analysis with a Zetasizer (Malvern).

### Cell Culture and Confocal Microscopy

Human skin microvascular endothelial cells (HMVEC, Sigma Aldrich) were cultured in CADMEC growth medium (Cell Applications), supplemented with 5% fetal bovine serum (FBS, Sigma Aldrich). Glass cover slides were placed in 24 well plates (Corning Costar) pre‐incubated with attachment factor solution (Cell Applications) at 37°C for 30 min. 5 × 10^5^ cells were seeded to each well and cultured to form an intact monolayer. After that, the cells were treated with AuNPs according to the required conditions. Then the cells were washed twice with Hanks’ Balanced Salt Solution (HBSS, Sigma Aldrich) and fixed in 4% paraformaldehyde (Sigma Aldrich) for 15 min. After that, immunofluorescent staining was performed to reveal the distribution and organization of VE‐cadherins and actin filaments. After washing with HBSS twice, the cells were blocked using 200 µL blocking buffer containing 0.1% saponin (Sigma Aldrich) and 5% horse serum (Sigma Aldrich) for 1 h at room temperature. Primary polyclone rabbit anti‐VE‐cadherin antibody (Abcam, ab33168, 1:400) was incubated with the cells at 4°C overnight, then donkey anti‐rabbit Alex 594 secondary antibody (Abcam, ab150076, 1:500) was used to conjugate with the primary antibody at room temperature for 2 h. Actin filaments were labelled by phalloidin‐iFluor 488 (Abcam, ab176753, 1:1000) at the same time. The cells were then stained by Hoechst 33 342 (Thermo Fisher Scientific) at room temperature for 5 min. After washing, the cover glasses were mounted with ProLong Gold (Thermo Fisher Scientific) and imaged using a confocal fluorescence microscope (SP8 LIGHTNING, Leica Microsystems). Semiquantitative image analysis was performed using ImageJ, where intercellular gaps in the immunofluorescence images were counted and the gap areas, amounts and feret diameters were measured by ImageJ.

### Reactive Oxygen Species (ROS)

≈8000 HMVECs/well were seeded into a 96‐well black plate and cultured overnight to reach 80% confluency. ROS detection was performed using an OxiSelect intracellular ROS detection kit. The cells were stained with H_2_DCFDA (20 µg mL^−1^) for 30 min and subsequently treated by AuNPs samples. ROS levels were then measured indirectly by the oxidation of nonfluorescent DCFDA to fluorescent DCF on a fluorescence microplate reader ClarioStar (BMG LABTECH), excited at 488 nm and detected at 535 nm. All samples were measured in triplicate. Untreated cells were used as negative control and H_2_O_2_ (200 × 10^−6^ m) as positive control.

### Microtome and Transmission Electron Microscopy

HMVECs were treated by AuNPs of three different sizes for 30 min and then washed twice by PBS. The cells were fixed by the mixture of 2.5% glutaraldehyde and 0.1 m sodium cacodylate overnight at 4°C, followed by osmium tetroxide polymerization (2%, 30 min at 20°C), uranyl acetate staining (1% overnight), and resin embedding (24 h, 60°C). Thick slices of 70 nm were prepared using a histo diamond knife (DiATOME, Switzerland) and a Leica EM UC6 microtome. The slices were placed on carbon‐coated copper grids (100 mesh, formvar film, ProSciTech), and images captured using a HITACHI HT7700 transmission electron microscopy operated at 80 kV.

### Transwell Insert Assays

HMVECs were first cultured on transwell inserts (polycarbonate membrane, 0.4 µm pore diameter; Corning Costar, USA) in a 24‐well plate until the formation of a monolayer (20 000 cells/well, 2 days). Cells were exposed to AuNPs of different sizes and concentrations, which were prepared in complete EndoGRO‐MV‐VEGF medium, for the duration of 1 h. In groups that were untreated with AuNPs, fresh culture medium was added. FITC–dextran (1 mg mL^−1^, 40 kDa; Sigma Aldrich) were included in all treatments. Subsequently, the solution at the bottom compartment of each well was collected and the fluorescence reading of the FITC‐dextran was measured by a microplate reader (Hidex, Finland) at wavelengths of 490/520 nm (excitation/emission). The degree of FITC‐dextran penetration was calculated by normalizing the fluorescence readings from treated group to its corresponding untreated control.

### Cell Viability Assay

HMVECs were seeded in a 96‐well plate and subsequently exposed to AuNPs (18, 30, and 70 nm; concentrations of 25 and 100 × 10^−6^ m) for the duration of 1 or 6 h. At the endpoint, alamarBlue reagent (Life Technology, USA) was prepared at recommended dilution from stock and cells were incubated with the mixture for 2 h. Fluorescence readings were measured at excitation/emission of 560/590 nm with a microplate reader (Hidex, Finland). Measurements from the negative control group were used to normalize against the readout from other treatment groups.

### Membrane Damage Assay

HMVECs were grown on 96‐well plates and exposed to the AuNPs for 1 or 6 h. The cells were washed with PBS subsequently and double stained for 30 min with cell permeant nucleic acid stain Hoescht 33 342 (1 µg mL^−1^; Life Technology, USA) and cell impermeable nucleic acid stain SYTOX Green (0.5 × 10^−6^ m; Life Technology, USA). The cells were once again washed with PBS, and the fluorescence signals were measured on a microplate reader (Hidex, Finland), at excitation/emission of 350/461 nm (Hoescht 33 342) and 495/530 nm (SYTOX Green) respectively. Fractions of cells with damaged membranes were determined by normalizing the SYTOX Green signal to the Hoescht signal. SYTOX Green readings from the control group were used to normalize the other groups’ readings.

### ROS Production Assay

HMVECs were cultured in 96‐well plates and then exposed to the AuNPs for 1 or 6 h. The cells were washed once with PBS and stained with a cocktail mixture of 1 × 10^−6^ m 2′,7′‐dichlorodihydrofluorescein diacetate (H_2_DCFDA; Merck, USA), a detector of ROS, as well as Hoescht 33 342 (1 µg mL^−1^) for 30 min. The cells were then washed with PBS. Fluorescence readings were measured on a microplate reader (Hidex, Finland) at excitation/emission of 495/527 nm (H_2_DCFDA) and 350/461 nm (Hoescht 33 342). ROS production levels in cells were determined by normalizing the H_2_DCFDA signal to the Hoescht signal. H_2_DCFDA signal readings from the control group were used to normalize the other groups’ readings.

### Assessments Involving Treatments Prior to AuNPs Exposure

For assays utilizing inhibitors (or other pre‐treatments), cultured HMVECs were exposed to the respective inhibitors, prepared in complete EndoGRO‐MV‐VEGF culture medium for 1 h. Subsequently, the treatment solution was replaced by a second solution containing both AuNPs and the inhibitor(s). Fresh culture medium containing only the inhibitor(s) acted as the second treatment for negative control groups. For the endocytosis experiments, endocytosis inhibitors methyl *β*‐cyclodextrin (M*β*CD, 5 × 10^−3^ m; Sigma Aldrich, USA) and monodansyl cadaverin (MDC, 10 × 10^−6^ m; Sigma Aldrich, USA) were employed. For experiments involving antioxidants, the antioxidant N‐acetyl cysteine (NAC, 10 × 10^−6^ m; Sigma Aldrich, USA) was utilized. For experiments investigating intracellular signaling under NanoEL, the Src family tyrosine kinase inhibitor PP1 (Sigma Aldrich, USA) was similarly used at 10 × 10^−6^ m in complete cell medium, as well as the Rho‐associated kinase inhibitor Y27632 (Sigma Aldrich, USA).

### Immunoblotting

HMVECs were cultured in 6‐cm cell culture dishes and exposed to the respective pre‐treatment solutions (if any) supplemented with inhibitors, followed by exposure to solutions containing the three sizes of Au NPs at 100 × 10^−6^ m for 1 h. Upon conclusion of the experiment, the cells were washed thrice with cold PBS and collected after cell lysis in Laemmli sample buffer (63 × 10^−3^ m Tris‐HCl, 2% sodium dodecyl sulphate (SDS), 10% glycerol, 1% 2‐mercaptoethanol and 0.0005% bromophenol blue; pH 6.8) which was supplemented with a mixture of 1% protease and phosphatase inhibitors (Sigma Aldrich, USA). The collected samples were subjected to gel electrophoresis using 10% resolving polyacrylamide gels (Mini Protean, Biorad, USA) and transferred onto nitrocellulose membranes. The membranes were subjected to 1 h of blocking with 5% bovine serum albumin (BSA) solution, before incubation in a solution of the relevant primary antibody at 4°C overnight. On the following day, the membranes were washed and incubated in a solution of the corresponding horseradish peroxidase (HRP)‐conjugated secondary antibody for 1 h. Protein bands on the membranes were visualized and captured through usage of the Immobilon Western Chemiluminescent HRP substrate kit (Merck, USA) in a chemiluminescence imaging setup (Syngene, UK). The images of protein bands were analyzed in a semi‐quantitative manner through ImageJ software, whereby bands yielded from treatment groups were all normalized against their respective control groups within each captured image. Throughout the immunoblotting process, Tris‐buffered saline with Tween 20 detergent (TBST; composed of: 150 × 10^−3^ m NaCl, 20 × 10^−3^ m Tris‐HCl, 0.1% Tween 20) was used for the preparation of blocking solution, antibody solutions as well as in washing steps. Primary antibodies were employed at dilution of 1:1000 and secondary HRP‐conjugated antibodies were employed at 1:3000. The complete list of antibodies utilized in this study are provided in Table S3 (Supporting Information).

### Vascular Leakiness Assay

For the ex vivo vascular leakiness assay, swine vessels were obtained from a local slaughterhouse in Chongqing. Three swines were used, the blood vessels of each pig were taken out and used for one experiment in 7 groups, and the experiment was repeated 3 times for three swines. Briefly, blood vessels of the coronary artery were cut transversely into individual pieces and placed in a commercial transwell chamber after removal of its original membranes. The blood vessel areas taken could surround the entire internal space of the transwell inserts. AuNPs of 18, 30, and 70 nm were added to the custom‐made swine vessel transwell device and incubated for 6 h. After the exposure, the AuNPs‐containing solution was discarded and then Evans blue dye (100 × 10^−3^ m) was added to each well for an additional 1 h. During the experiment, transwell inserts were placed in a 24 well plate, and then the 24 well plate was placed at 37°C for static culture. Finally, the fluorescence signal from the lower compartment of the transwell was quantified at 624 nm with a microplate reader. Readout from the negative control group was used for normalization.

### Discrete Molecular Dynamics

Discrete molecular dynamics (DMD) is a special category of molecular dynamics (MD) where the conventional MD force field is remodeled as discrete step functions.^[^ ^21^ ^]^ DMD simulations have been widely applied to biomolecular studies such as protein aggregation,^[^ ^22^ ^]^ protein structure and dynamics,^[^ ^23^ ^]^ and protein‐nanoparticle interactions.^[^ ^24^ ^]^ Here, the bonded (i.e., bonds, bond angle, and dihedral angle) and non‐bonded terms (van der Waals, electrostatic, solvation, and hydrogen bonds) comprised the inter‐atomic potential for DMD simulations. Among the non‐bonded terms, the solvation term was employed by the EEF1 implicit solvent model determined by Lazaridis and Karplus^[^ ^25^ ^]^ and the hydrogen bond term was modeled with the reaction‐like algorithm.^[^ ^26^ ^]^ CHARMM forcefield^[^ ^27^ ^]^ and Debye‐Hückel approximation were applied to van der Waals and the electrostatic terms in non‐bonded parameters.

### DMD Simulations for AuNP‐Cadherin Dimer Binding

Cadherins are important proteins for both intra and inter cell to cell adhesion through the trans and cis interactions. Here, the trans interaction was formed by the extracellular domain of cadherin from two opposing cells and the interface of trans‐dimer was stabilized by the domain‐swapped region in EC1 domains. The EC1 cadherin domains were used from the cryo‐EM model of EC12 cadherin dimer to identify the stability of the EC1 cadherin dimer (PDB ID: 3PPE).^[^ ^1a^ ^]^ The domain‐swapping of the N‐terminal fragment (i.e., residues 1–5) as well as the Ca^2+^‐coordination of the loops (i.e., residues Glu11, Asp62, Glu64, Asp96, and Asp99) have been identified to stabilize the VE‐cadherin.^[^ ^1a^ ^]^ To satisfy these conditions, the Gō‐potential and bond constraints were applied on domain‐swapped and the residue related to calcium ion binding, respectively (Figure 5A). Subsequently, three different sized AuNPs of 1, 2, and 3 nm in diameter (Figure 5B) were constructed to investigate their binding to the EC1 cadherin dimer. All AuNPs were randomly distributed near the EC1 cadherin dimer at least 12 Å away in a 12^3^ nm^3^ cubic box. During the binding simulations, the backbone of EC1 dimer was constrained and sidechains were freely interacted with the AuNPs. 30 independent DMD simulations lasted for 50 ns, and an accumulative 1.5 µs DMD simulations were carried out after gradual temperature relaxations from 250 to 300 K. 50 fs/step of the unit simulation time and 1 kcal mol^−1^ of corresponding energy were applied. During the binding simulations of AuNP with the EC1 and EC12 cadherin dimers, a temperature of 300 K was maintained with Anderson's thermostat. DMD simulations with different initial configurations as well as velocities and energy relaxation were performed to avoid a biased potential energy. After the binding simulations, the binding frequencies of the AuNPs with the EC1 cadherin dimer were computed except for the first 20 ns of simulations to avoid a potential bias. To calculate the binding frequency, a cutoff distance of 0.65 nm was defined to obtain an atomistic contact between the AuNPs and the EC1 cadherin dimer.

### Steered Discrete Molecular Dynamics (sDMD) Simulations

After the binding simulation of AuNPs with the EC1 and EC12 cadherin dimer, constant force‐pulling was performed in silico experiments on the cadherin‐AuNP complexes to investigate the EC1‐EC1 cadherin dimer stability through steered molecular dynamics (sDMD) simulations. sDMD simulations have been used for identifying the protein‐ligand binding^[^ ^28^ ^]^ and protein unfolding induced by mechanical forces. This approach mimics the experimental techniques of atomic force microscopy (AFM) and optical tweezers for measuring biomolecular forces and characterizing biomolecules based on their response to a constant velocity or force. The previous study effectively determined the critical forces and free energies of polyamidoamine (PAMAM)−protein complexes through sDMD simulations.^[^ ^28^ ^]^ Prior to the sDMD simulations, one of the cadherin domain backbones of the EC1 dimer was immobilized and the other domain was given to flexible states (Figure 5E). During the sDMD simulations, the flexible domain from EC1 cadherin dimer was pulled along the EC1 to EC2 direction. Subsequently, counter ions were distributed near the AuNP‐EC1 cadherin complex and the initial atomic velocity of all system was randomized. To ensure sufficient sampling, 30 cases of independent sDMD simulations each for 100 ns was carried out. During the sDMD simulations, constant forces were applied to the flexible region of the EC1 cadherin dimer. 10 pN of interval force was assigned to the force range of 0 to 60 pN. Same as the simulation for AuNP and EC1 cadherin binding, a temperature of 300 K was maintained with Anderson's thermostat and 50 fs/step of the unit simulation time and 1 kcal mol^−1^ of corresponding energy were considered.

### Measurement of EC1 Cadherin Dimer Angle

During the dimerization of the various species of cadherin, the cadherin dimer can exist in two states such as the intermediate (x‐dimer, PDB ID 4ZT1) and the domain‐swapped dimer (s‐dimer) with distinct dimer angles. The crystal structure of x‐dimer was identified from human E‐cadherin.^[^ ^1^, ^15^ ^]^ For identifying both the x‐dimer and s‐dimer angles, two vectors of the G‐strands in the EC1 dimers were considered. The notation of *β*‐strands was adapted according to the canonical Ig fold of the EC1 domain.^[^ ^1a^ ^]^ To calculate the s‐dimer angle, the dot product of the vector between the 88^th^ and 95^th^ residues of the EC1 cadherin dimer from VE‐cadherin was computed, to acquire the dimer angle of the EC1 cadherin dimer with and without the AuNP for the first 30 ns sDMD simulation before the dimer started to dissociate (Figure 5I, upper panel). For reference, a crystal structure of the s‐dimer of the EC1 domains of VE‐cadherin^[^ ^1a^ ^]^ is ≈85° and the dimer angle of the x‐dimer from the crystal structure of E‐cadheri^[^ ^13a^ ^]^ is ≈0.5°. The x‐dimer angle was calculated as the dot product of the G‐strands vector between the 92^th^ and 99^th^ residues (Figure 5I, lower panel). The notation of the *β*‐strands was adapted from the previous study.^[^ ^1a^ ^]^

### Mesoscale Modelling of Cadherin Pair Dissociation

According to Bell's mode,^[^ ^29^ ^]^ the dissociation rate k_off_ of a single cadherin pair increases exponentially with the applied force *f*

(1) $$k_{\mathrm{off}}=\mathrm{Aexp}\left(-\frac{\Delta G-fx_{b}}{k_{B}T}\right)$$

where A is the pre‐exponential coefficient, k_B_ is Boltzmann's constant and *T* is the absolute temperature. ΔG is the energy barrier between ground state and transition state to dissociate a single cadherin pair, and x_b_ is the projection of the transition state along the force vector. The dissociation rate can be simplified as $k_{\mathrm{off}}{\;=k}_{\mathrm{off}}^{0}\;\exp\left(f/F_{b}\right),$ where $k_{\mathrm{off}}^{0}=\mathrm{Aexp}\left(-\Delta G/k_{B}T\right)$ is the intrinsic dissociation rate of the unstressed cadherin pair. For strongly bound cadherins pairs, $k_{\mathrm{off}}^{0}$ ranges from 1  × 10^−5^ to 1  × 10^−4^ s^−1^.^[^ ^17^ ^]^ F_b_ = k_B_T/x_b_  is the thermal force. Atomic force microscope measurements showed that F_b_ ≈ 5 pN for cadherins.^[^ ^17^, ^30^ ^]^ According to the sDMD simulations, the AuNPs could reduce the energy barrier to dissociate the cadherin pair. Thus, the dissociation rate of the cadherin pair bound with the AuNP is

(2) $$k_{\mathrm{off}}{=k}_{\mathrm{off}}^{0}\;\exp\left(\frac{\Delta \Delta G}{k_{B}T}\right)\exp\left(\frac{f}{F_{b}}\right)$$

where ΔΔG is the reduction of energy barrier induced by the AuNPs.

For the leakiness of endothelial cells connected by multiple cadherin pairs, the rebinding of unbound cadherin pairs when two cadherins are in close proximity for a sufficient amount of time should be taken into consideration. The rebinding rate k_on_ was assumed to depend on the separation between two unbound cadherins. The separations between cadherins were induced by the deformation of cells and elongation of cadherin pairs under pulling forces. To generalize the rebinding process, the reaction between two cadherins tethered to the cell wall was modeled by a linear spring with stiffness k_CD_ and rest length l_CD_ (Figure 6A).^[^ ^31^ ^]^ At a given separation *d*, the probability density function *P* for the receptor to have a displacement *u* is

(3) $$P\;\left(u\right)=\frac{1}{Z}\;\exp\left(-\frac{k_{\mathrm{CD}}u^{2}}{2k_{B}T}\right)$$

where the partition function Z satisfies the normalization condition $\int_{-l_{\mathrm{CD}}}^{d-l_{\mathrm{CD}}}P\left(u\right)\mathrm{du}=\;1.$ ^[^ ^32^ ^]^ The probability that two cadherins come within a reacting radius l_bind_ of the binding site is then $p\;=\frac{\;l_{\mathrm{bind}}}{Z}\;\exp\left(-\frac{k_{\mathrm{CD}}{\left(d-l_{\mathrm{CD}}\right)}^{2}}{2k_{B}T}\right).$ Hence, the rebinding rate is

(4) $$k_{\mathrm{on}}{=k}_{\mathrm{on}}^{0}\;\frac{\;l_{\mathrm{bind}}}{Z}\exp\left(-\frac{k_{\mathrm{CD}}{\left(d-l_{\mathrm{CD}}\right)}^{2}}{2k_{B}T}\right)$$

where $k_{\mathrm{on}}^{0}$ is the reaction rate between the cadherin pairs. In the simulations, l_bind_ =  1 nm, l_CD_ =  12 nm, k_CD_ =  4  pN nm^−1^ and $k_{\mathrm{on}}^{0}/k_{\mathrm{off}}^{0}=5\;\times 10^{5}$ was set.^[^ ^32^ ^]^

The deformation of cells caused by the pulling force on cadherins was modeled by elastic media subjected to discretely distributed tensile stress on each cadherin pair. The tensile stress on cadherin pair *i* is q_i_ = f_i_/*π*a^2^ , where a  = 1.5 nm is the radius of cadherin pair. The displacement w_ij_ at x_i_ induced by cadherin at x_j_ is given by

(5) $$\begin{array}{c} w_{\mathrm{ij}}=\frac{4q_{i}r_{\mathrm{ij}}}{\pi E^{*}}\;\left[\int_{0}^{\frac{\pi }{2}}{\left(1-\frac{a^{2}}{{r_{\mathrm{ij}}}^{2}}\mathrm{si}n^{2}\theta \right)}^{\frac{1}{2}}d\theta -\left(1-\frac{a^{2}}{{r_{\mathrm{ij}}}^{2}}\right)\int_{0}^{\frac{\pi }{2}}{\left(1-\frac{a^{2}}{{r_{\mathrm{ij}}}^{2}}\mathrm{si}n^{2}\theta \right)}^{-\frac{1}{2}}d\theta \right] \end{array}$$

where r_ij_ is the distance between the x_i_ and x_j_.^[^ ^33^ ^]^ The reduced elastic modulus E* = E/2(1−*μ* ^2^), where E is elastic modulus and *μ* is the Poisson's ratio of cell. In the simulations, E* =  3  × 10^3^ kPa was set.^[^ ^34^ ^]^ Then the total displacement w_i_ at x_i_ is $w_{i}=\sum_{j=1}^{n}w_{\mathrm{ij}}$, where n is the total number of bound bonds.

The conditions of interface compatibility and global force balance are w_i_+u_i_+  l_CD_ =  h and $\sum_{i\;=\;1}^{n}f_{i}=\mathrm{qN}b^{2},$ where h is the unknown total cell–cell surface separation, q is the tensile stress subjected by endothelial cells, N is the total number of cadherin pairs, and b is the spacing distance between cadherins. Averaged tensile force per cadherin pair $f\;=\sum_{i=1}^{n}f_{i}/N\;$was used in analyses in consistent with the sDMD results. In the simulations, 40 cadherin pairs with periodic boundary conditions enforced to eliminate the size effects were considered. The spacing distance between cadherins is b  =  5 nm, corresponding to the surface density of cadherins as 4  × 10^4^ *µ*m^−2^.^[^ ^32a^ ^]^ Once the n + 1 unknowns (F_1_,F_2_,⋅⋅⋅,F_n_,h) are solved, the surface separation distance between the two elastic media can be calculated by d_i_ =  h‐w_i_.

The forces on bound cadherin pairs and surface separations at unbound cadherin pairs were used to compute the respective dissociation and rebinding rates for any instantaneous conformation. Monte Carlo‐based first reaction method was adopted to simulate the evolution of cadherin pair states.^[^ ^35^ ^]^ Briefly, a series of independent random numbers *η*_1_, *η*_2_, *η*_3_⋅⋅⋅*η*_N_ uniformly distributed over the interval [0, 1] were generated at each simulation step. The time for next reaction of cadherin pair *i* was d *τ*_i_ = −ln*η*_i_/k_i_, where k_i_ =k_off_ if the cadherin pair was current bound and k_i_  =  k_on_ if the cadherin pair was currently unbound. Based on the first reaction method, the time for the next reaction was chosen as the minimum of d*τ*_i_ and the location for the next event was identified to the cadherin pair where d*τ*_i_ was chosen. Then, the chosen cadherin pair was set to be unbound if it was currently bound or set to be bound if it was currently unbound. After the change of cadherin pair states, the force distribution and surface separation between cells were updated, which were then used to determine the subsequent reactions. The above procedure was repeated until all the cadherin pairs were unbound and the total elapsing time was recorded as the lifetime of cadherin clusters. For each system, 50 independent simulations with different random number generator seeds were performed and each simulation run for 10^5^ steps unless all the cadherin pairs were unbound.

### Statistical Analysis

The sizes of the AuNPs determined by TEM imaging were represented by mean ± SD. The in vitro assays of cell viability, membrane damage and ROS production, in vitro and in vivo transwell assays, semi‐quantitative analyses of visualized immunoblots and actin network were derived from at least three biologically independent samples (*n* = 3). The extent of endothelial leakiness was expressed by gap area and distribution, which were derived from the images using trainable Weka segmentation plugin in ImageJ software. Data collected were normalized against each group's negative control and presented as mean ± SD. One‐way ANOVA and two‐way ANOVA with Tukey's multiple comparison tests were performed as indicated in the respective figure captions, using the software GraphPad Prism 8. In all scenarios, statistical significance was the result of two‐tailed testing, where symbols *^, #,^ *^&^* all represent *P* < 0.01 while **^, ##, &&^ all denote *P* < 0.001 between the various compared groups. All analyses of images were performed in ImageJ software, as described in the respective figure captions.

## Conflict of Interest

The authors declare no conflict of interest.

## Supporting information

Supporting Information

## Data Availability

The data that support the findings of this study are available from the corresponding author upon reasonable request.
