# Supplementary material for: A Framework of Paracellular Transport via Nanoparticles‐Induced Endothelial Leakiness
Source: Adv Sci (Weinh). 2021 Sep 8;8(21):2102519. doi: 10.1002/advs.202102519 (PMC8564447; doi:10.1002/advs.202102519)
Supplement: Supplementary file 1 — Supporting Information [file ADVS-8-2102519-s001.pdf]

WILEY-VCH

© 2021 Wiley-VCH GmbH

ADVANCED SCIENCE
Open Access

Supporting Information

for *Adv. Sci.,* DOI: 10.1002/advs.202102519

A Framework of Paracellular Transport via Nanoparticles-Induced Endothelial Leakiness

Myeongsang Lee,[†] *Nengyi Ni,*[†] *Huayuan Tang,*[†] *Yuhuan Li,*[†] *Wei Wei, Aleksandr Kakinen, Xulin Wan, Thomas P. Davis, Yang Song,*[*] *David Tai Leong,*[*] *Feng Ding*[*] *and Pu Chun Ke*[*]

Supporting Information

A Framework of Paracellular Transport via Nanoparticles-Induced Endothelial Leakiness

Myeongsang Lee,† Nengyi Ni,† Huayuan Tang,† Yuhuan Li,† Wei Wei, Aleksandr Kakinen, Xulin Wan, Thomas P. Davis, Yang Song, David Tai Leong,* Feng Ding* and Pu Chun Ke**

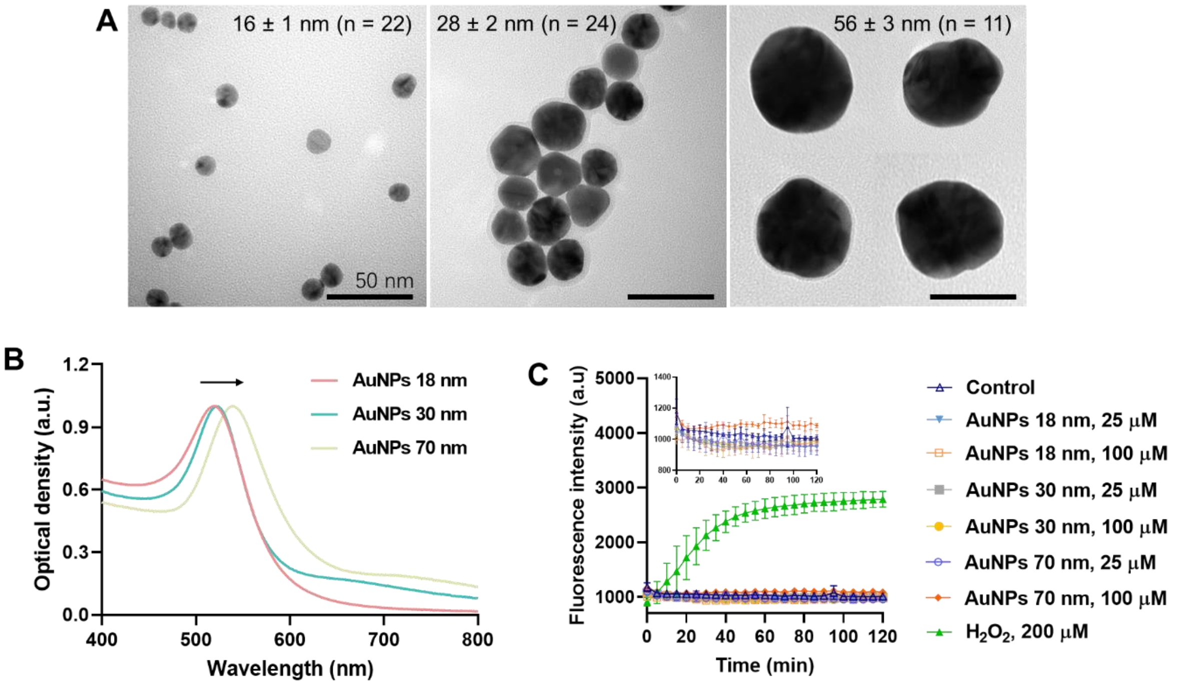

Figure S1. Characterizations of the AuNPs used in the study. (A) Transmission electron microscopy imaging of AuNPs of 18 nm (left), 30 nm (middle) and 70 nm (right) in nominal size. Scale bars: 50 nm. (B) The peak absorbance wavelength increased with the increasing size of the AuNPs. (C) Reactive oxygen species (ROS) generated in HMVECs over 0 to 120 min by AuNPs of different sizes and concentrations. The inset is a zoomed-in plot for the samples without H_2O_2. Data are shown as the mean ± SD (n=3) and statistical analysis was performed through two-tailed Student's *t*-test. Compared with the control, there was no significant difference observed in different groups of AuNPs.

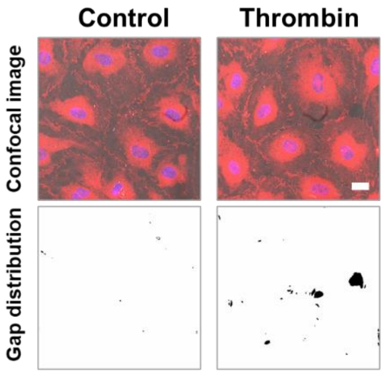

Figure S2. Intact endothelial cell monolayer and endothelial leakiness induced by thrombin (3 U/mL) in HMVECs. The images with black dots on a white background revealed the gaps' distribution which were performed by the trainable Weka segmentation plugin in ImageJ software. Scale bar: 20 μm.

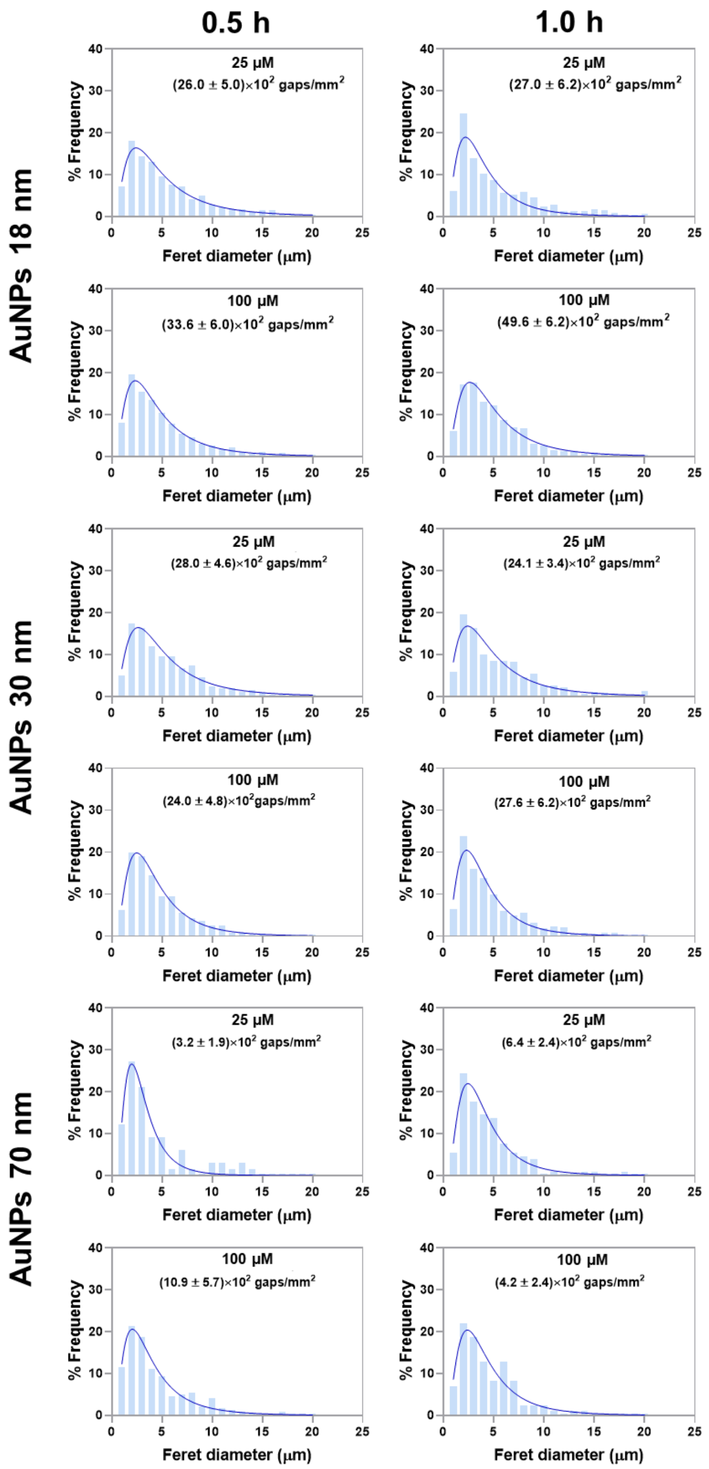

Figure S3. Gap size and number analyses for AuNPs-induced endothelial leakiness in HMVECs. The analyses were performed by ImageJ software and displayed through the frequency plot of the gaps' feret diameter. The predominant feret diameter of the gaps was around 2 μm. The number of gaps in each group was enlisted as insets in each plot.

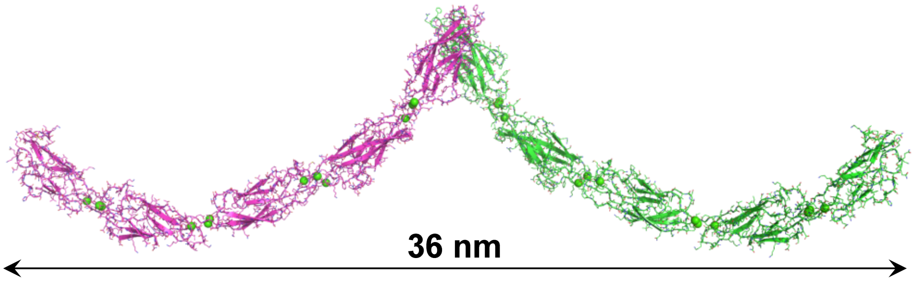

Figure S4. The extracellular domains of a VE-cadherin dimer (PDB ID: 1Q55) has a length of ~36 nm. Here, the dimer is shown in cartoon with one chain in red and the other chain green. Calcium atoms are shown as spheres. NPs with sizes larger than 36 nm will have a greater difficulty to enter and be aligned to disrupt the homophilic interactions of VE-cadherins.

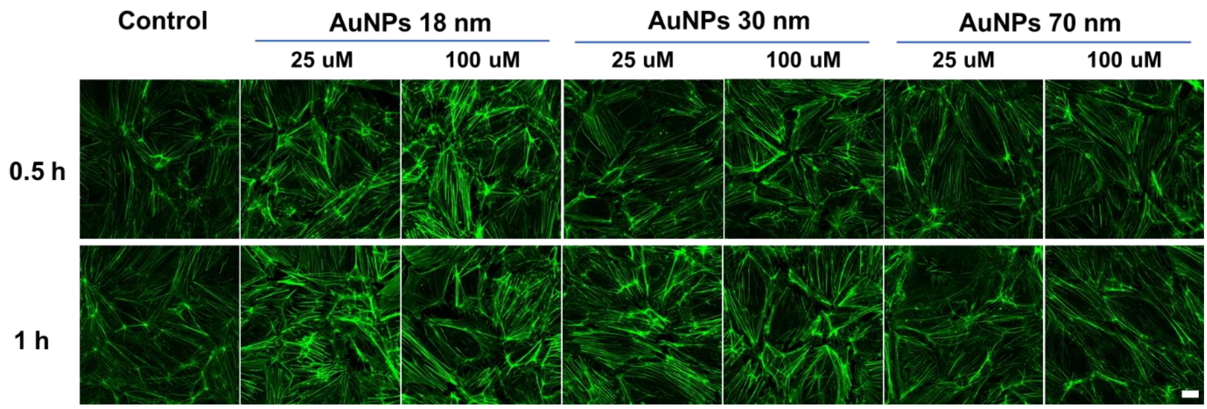

Figure S5. AuNPs induced actin reorganization in HMVEC cells. Confocal fluorescence microscopy observed the arrangement of actin in the presence of different sizes (18, 30 and 70 nm) and concentrations (25 and 100 μM) of AuNPs upon 0.5 h and 1 h treatments. Scale bar: 20 μm.

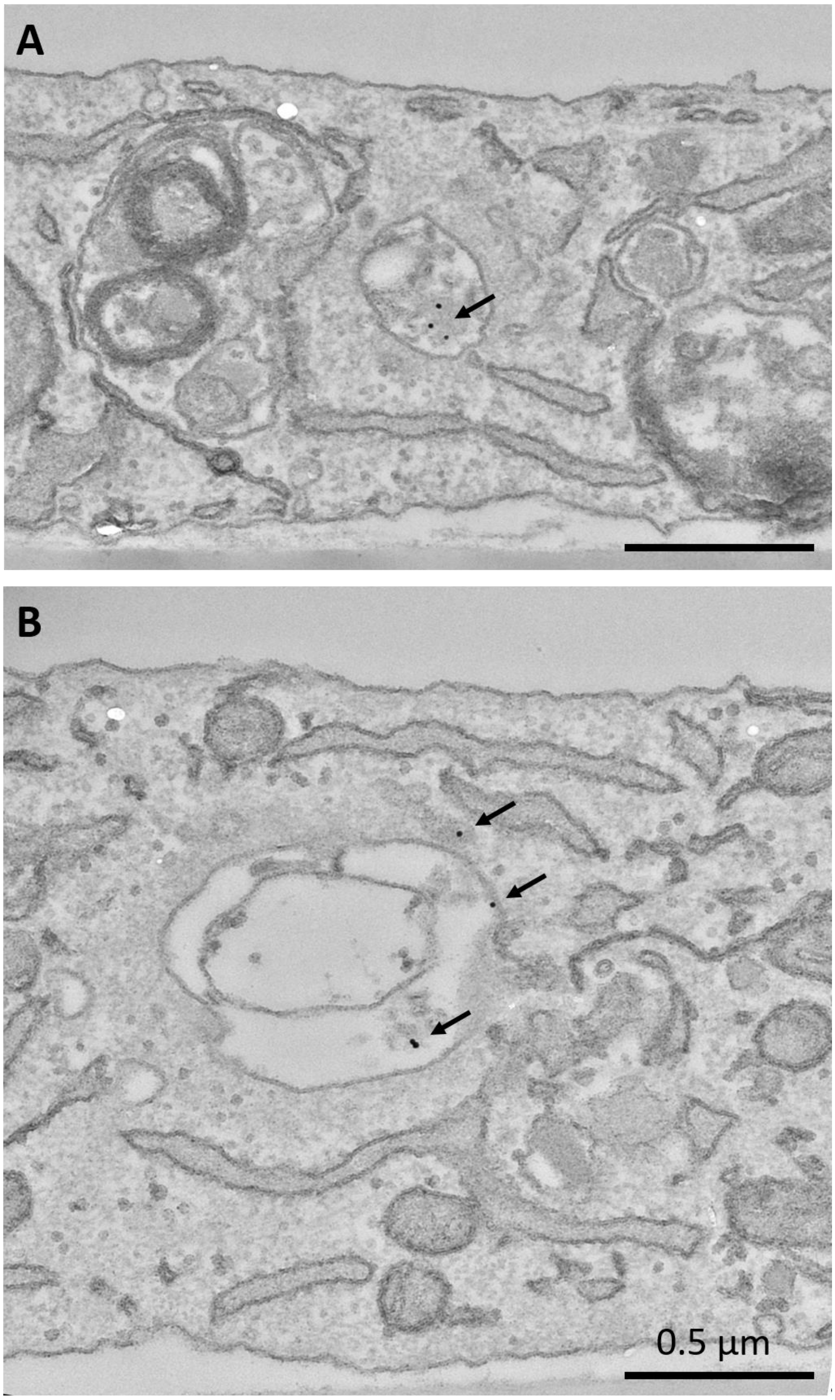

Figure S6. (A, B) Microtome transmission electron microscopy imaging revealed traces of endocytosis of AuNPs (18 nm in size) by HMVECs.

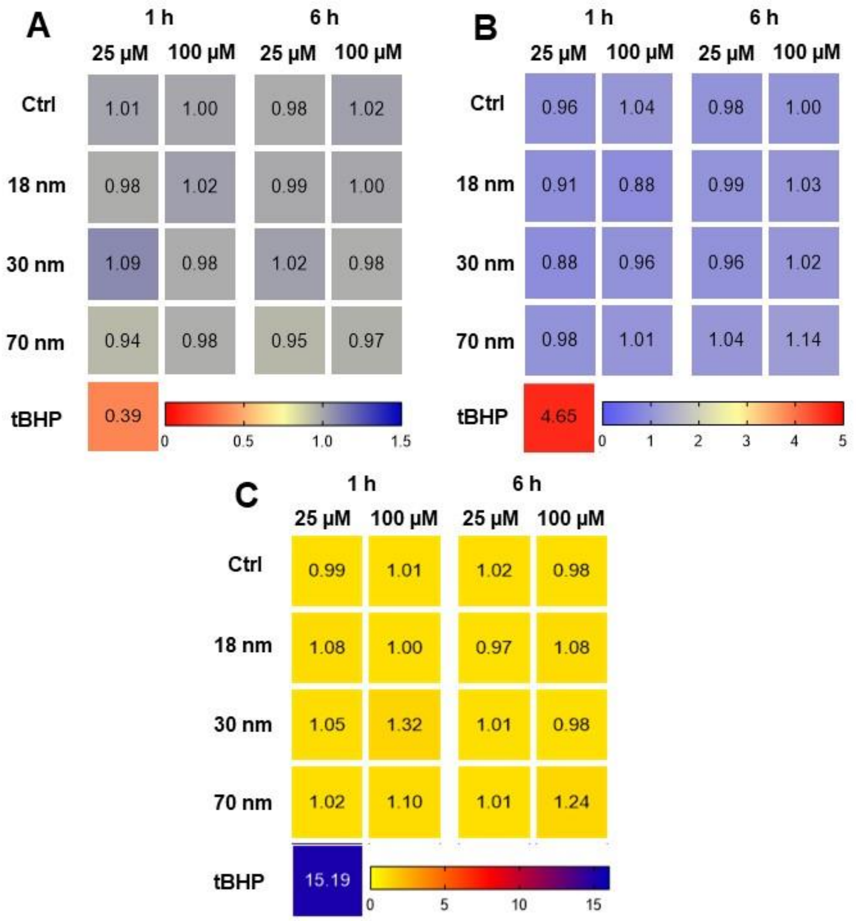

Figure S7. AuNPs-induced endothelial leakiness was independent of any decrease in cell viability, increase in membrane damage or increase in oxidative stress. (A) No significant changes were observed in HMVEC viability after treatment with different sized AuNPs (18, 30 and 70 nm in diameter; concentrations of 25 µM and 100 µM) for the exposure of 1 h or 6 h. (B) Similarly, no significant increase in membrane damage was observed in AuNPs-treated HMVEC at 1 h. tert-Butyl hydroperoxide (tBHP; 500 µM, 6 h) was used as positive control. (C) No significant increase in ROS production was observed in HMVEC after treatment with the AuNPs at 1 h or 6 h. tBHP (500 µM, 1 h) served as positive control. Heat map data represent the mean of three biologically independent samples. Statistical analyses were performed via two-way ANOVA with Tukey's multiple comparison tests, to yield no significant differences between treatment groups and respective control groups.

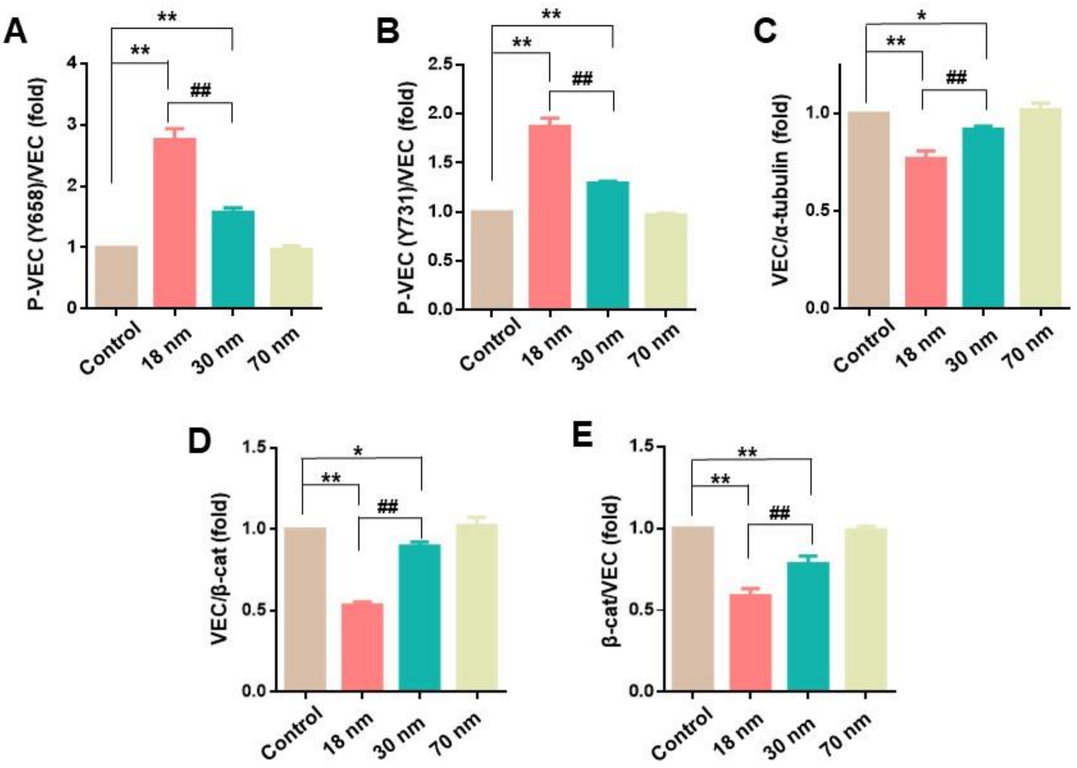

Figure S8. Endothelial leakiness induced by AuNPs required activation of VE-cadherin signaling. (A-C) Semi-quantitative analyses were performed on the levels of VE-cadherin (VEC), P-VEC(Y658) and P-VEC(Y731) detected via immunoblotting of HMVECs exposed to different AuNPs (100 µM, 1 h). (D-E) Semi-quantitative analysis was performed on the levels of VEC detected after β-cat immunoprecipitation of different AuNPs-treated HMVEC groups (100 µM, 1 h). Similarly, analysis was performed on the levels of β-cat detected when VEC was the immunoprecipitant. Data are mean ± SD, derived from visualized immunoblots of biologically independent samples (n=3), which were semi-quantitatively analyzed using ImageJ for protein levels. Statistical tests conducted were one-way ANOVA tests, where * represents $P < 0.01$ and $^{**,\#\#}$ represents $P < 0.001$, between the compared groups.

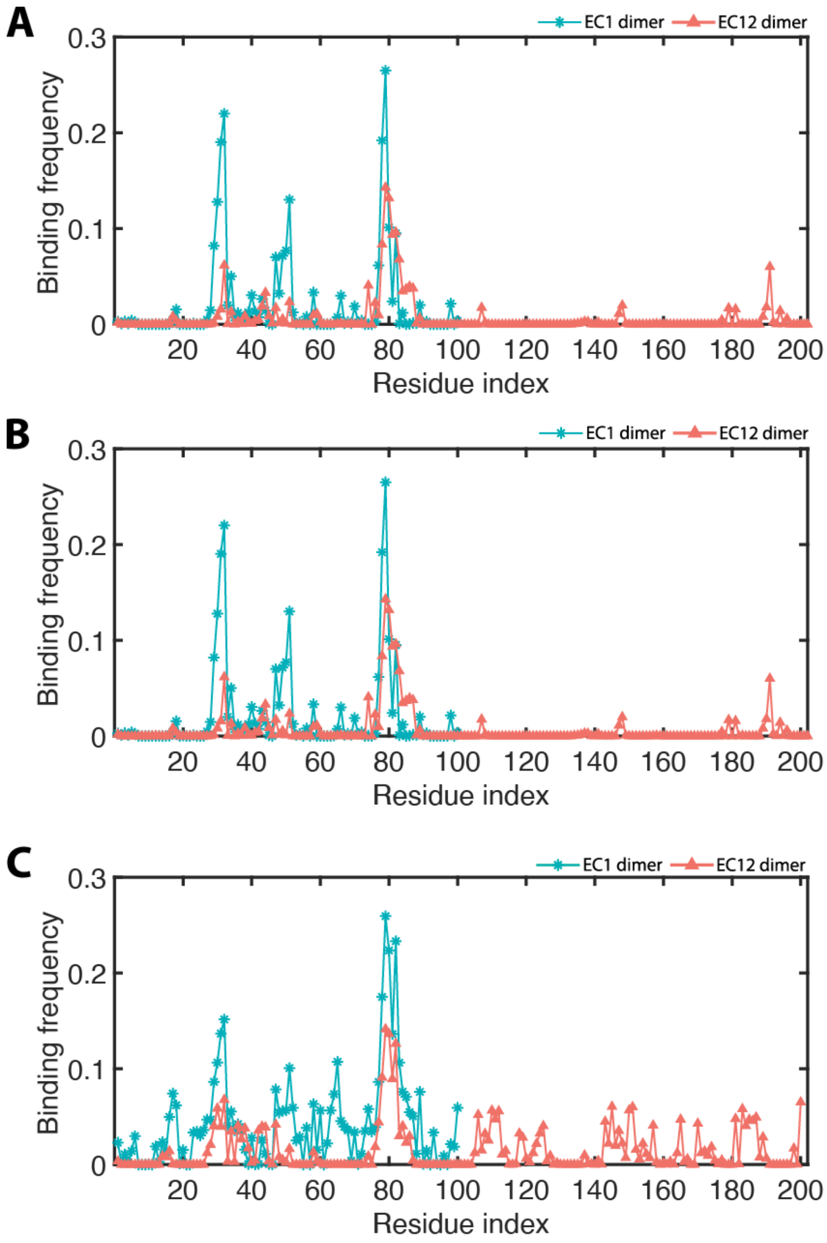

Figure S9. Binding frequency comparison of AuNPs with EC1 and EC12 cadherin dimer. Binding frequency of the AuNPs with EC1 dimer and EC12 dimer were marked as stars and triangles, respectively. Binding frequency of (A) 1 nm of AuNP, (B) 2 nm of AuNP, and (C) 3 nm of AuNP with EC1 and EC12 cadherin dimer.

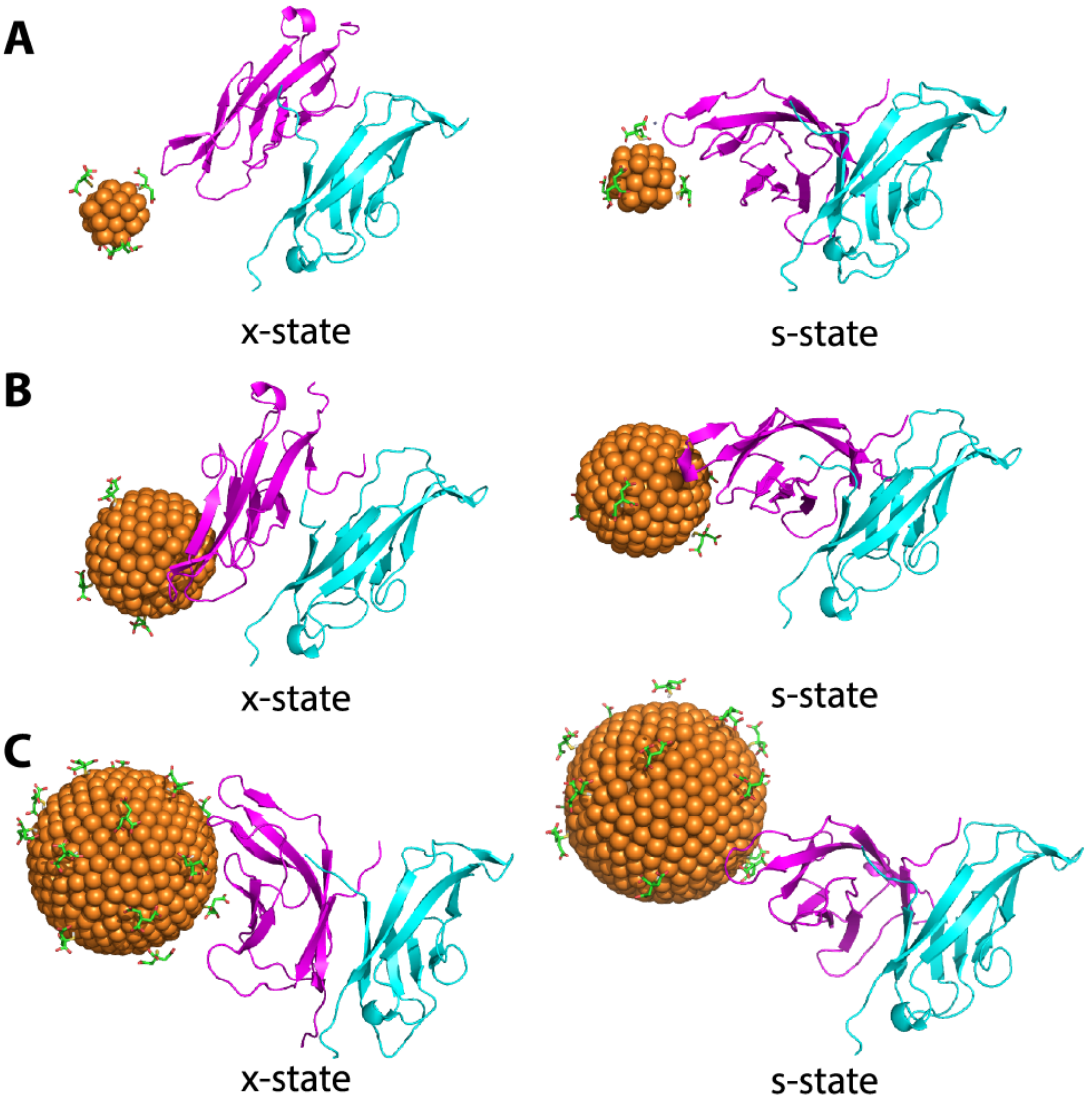

Figure S10. Schematic of two different states of cadherin in the presence of different sized AuNPs. Two different states of the EC1 cadherin dimer can be classified as the x-dimer and s-dimer. The x-dimer and s-dimer represent the intermediate and domain-swapped states of the cadherin dimer, respectively. The cyan and magenta colors indicate the immobilized cadherin domain and the flexible domain of the EC1 cadherin dimer. Two different states of the EC1 cadherin dimer with (A) 1 nm of AuNP, (B) 2 nm of AuNP, and (C) 3 nm of AuNP.

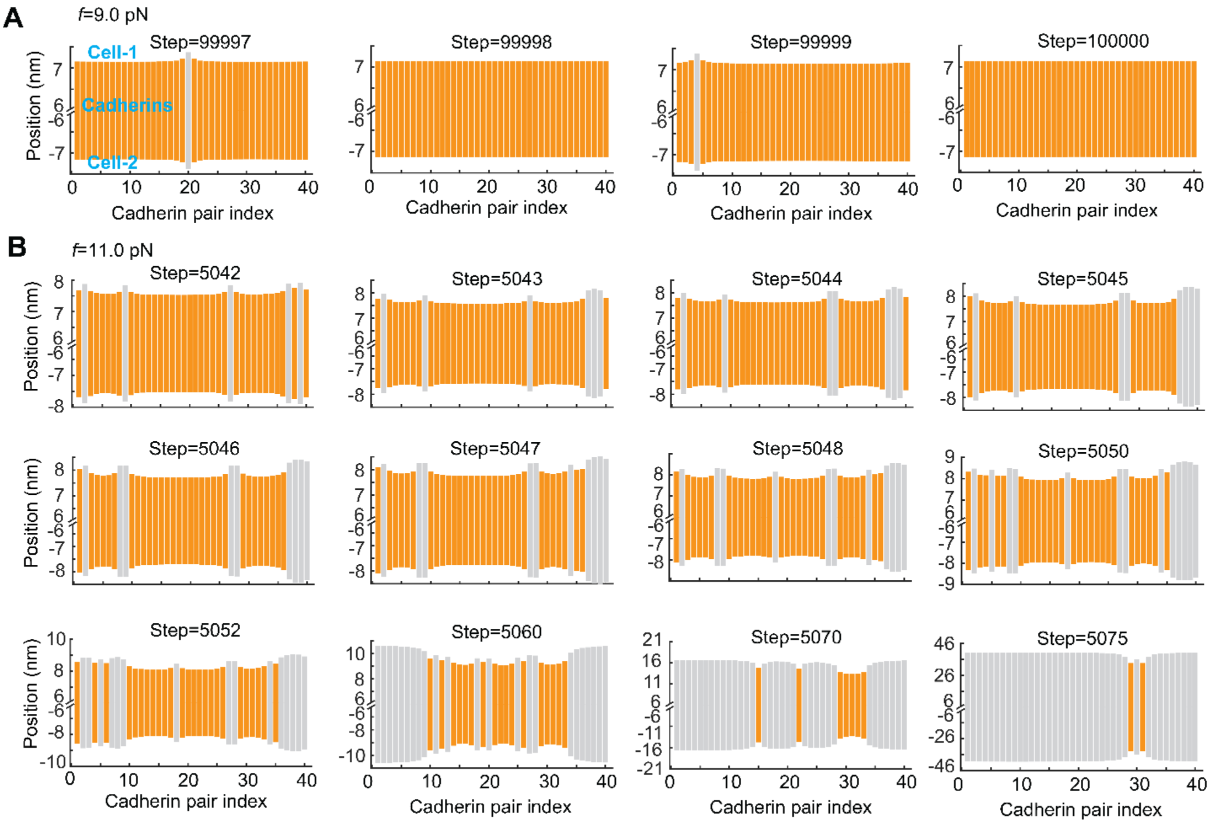

Figure S11. Time evolution of cadherin pair states and deformations in the absence of AuNPs. Positions were measured from the symmetric plane parallel to cell surfaces when all the cadherin pairs were in bound states. Cadherin pairs in bound and unbound states were colored in orange and gray, respectively. (A) $f = 9.0$ pN; (B) $f = 11.0$ pN.

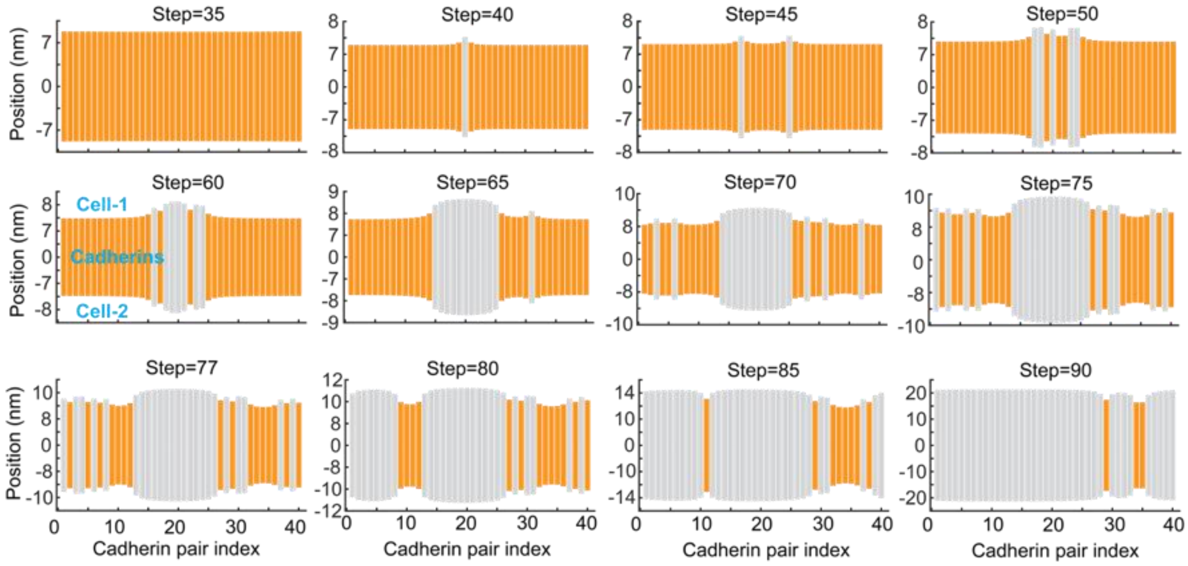

Figure S12. Time evolution of cadherin pair states and deformations when the AuNPs were localized in the center region of cadherin pairs with the AuNP/cadherin ratio=0.25 and tensile force $f = 10.0$ pN. Positions were measured from the symmetric plane parallel to cell surfaces when all the cadherin pairs were in bound states. Cadherin pairs in bound and unbound states were colored in orange and gray, respectively.

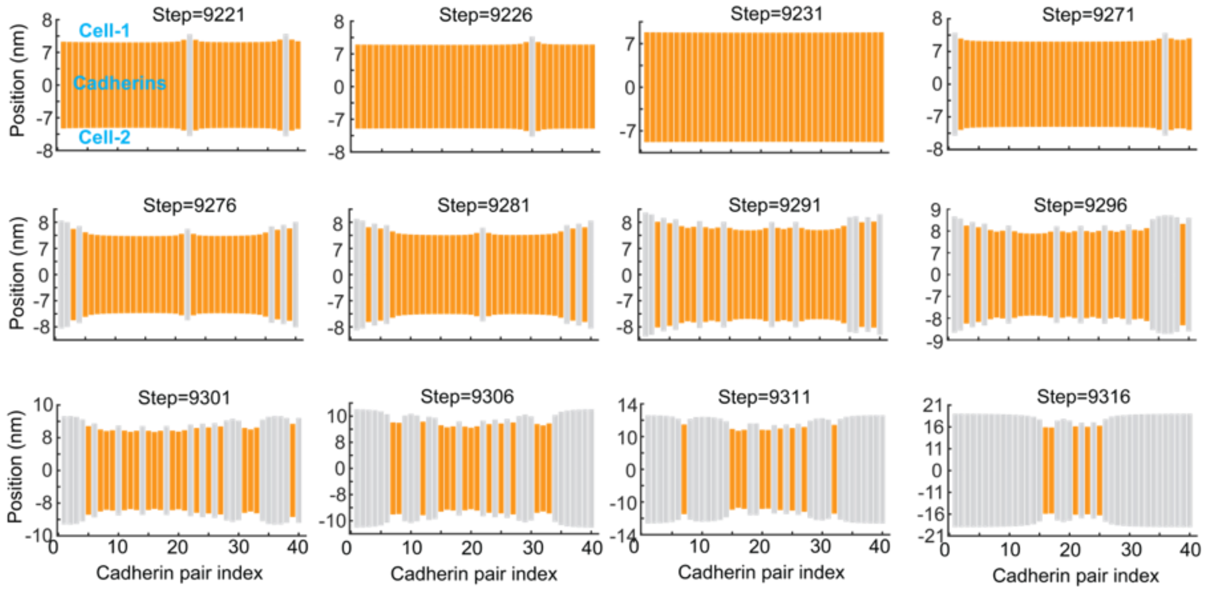

Figure S13. Time evolution of cadherin pair states and deformations when the AuNPs were uniformly distributed among cadherin pairs with the AuNP/cadherin ratio=0.25 and tensile force $f = 10.0$ pN. Positions were measured from the symmetric plane parallel to cell surfaces when all the cadherin pairs were in bound states. Cadherin pairs in bound and unbound states were colored in orange and gray, respectively.

Table S1. Hydrodynamic diameter and ζ-potential measurement of AuNPs in ultrapure water and complete cell medium by DLS.

	**Ultrapure water**		**Complete cell medium**	
**AuNPs**	**Hydrodynamic size (nm)**	**ζ-potential (mV)**	**Hydrodynamic size (nm)**	**ζ-potential (mV)**
18 nm	16.8 ± 1.4	-39.9 ± 5.7	32.6 ± 4.9	-12.9 ± 1.9
30 nm	38.7 ± 1.2	-41.7 ± 3.9	55.9 ± 4.7	-12.5 ± 1.9
70 nm	68.4 ± 5.4	-26.7 ± 1.5	92.9 ± 2.3	-15.4 ± 3.7

Table S2. Gap area analysis corresponding to the confocal images of endothelial leakiness induced by AuNPs.

**Samples/ Gap area (%)**	**0.5 h**		**1.0 h**	
	**25 µM**	**100 µM**	**25 µM**	**100 µM**
AuNPs 18 nm	3.75 ± 0.80	4.85 ± 1.03	4.55 ± 0.77	5.48 ± 0.60
AuNPs 30 nm	3.18 ± 0.67	2.56 ± 0.52	3.23 ± 0.85	3.24 ± 0.61
AuNPs 70 nm	0.19 ± 0.07	0.81 ± 0.34	0.48 ± 0.27	0.35 ± 0.40

Table S3. Complete list of antibodies employed in the immunoblotting experiments.

**Antibody**	**Product origin**
Anti-phospho VE-cadherin (Y658)	Thermo Fisher Scientific
Anti-phospho VE-cadherin (Y731)	Thermo Fisher Scientific
Anti-VE-cadherin	Cell Signaling Technology
Anti-β-catenin	Cell Signaling Technology
Anti-α-tubulin	Cell Signaling Technology
HRP-conjugated mouse anti-rabbit	Cell Signaling Technology
